# Dynamic strain determination using fibre-optic cables allows imaging of seismological and structural features

**DOI:** 10.1038/s41467-018-04860-y

**Published:** 2018-07-03

**Authors:** Philippe Jousset, Thomas Reinsch, Trond Ryberg, Hanna Blanck, Andy Clarke, Rufat Aghayev, Gylfi P. Hersir, Jan Henninges, Michael Weber, Charlotte M. Krawczyk

**Affiliations:** 1 GFZ German Research Centre for Geosciences, Telegrafenberg, Potsdam, 14473 Germany; 2 ÍSOR Iceland GeoSurvey, Grensásvegi 9, Reykjavik, 108 Iceland; 3 Silixa Ltd., Silixa House, 230 Centennial Park, Centennial Avenue, Elstree, WD6 3SN UK; 4 Institute of Earth and Environmental Science, University of Potsdam, Potsdam, 14476 Germany; 5 Institute for Applied Geosciences, Technical University Berlin, Ernst-Reuter-Platz 1, Berlin, 10587 Germany

## Abstract

Natural hazard prediction and efficient crust exploration require dense seismic observations both in time and space. Seismological techniques provide ground-motion data, whose accuracy depends on sensor characteristics and spatial distribution. Here we demonstrate that dynamic strain determination is possible with conventional fibre-optic cables deployed for telecommunication. Extending recently distributed acoustic sensing (DAS) studies, we present high resolution spatially un-aliased broadband strain data. We recorded seismic signals from natural and man-made sources with 4-m spacing along a 15-km-long fibre-optic cable layout on Reykjanes Peninsula, SW-Iceland. We identify with unprecedented resolution structural features such as normal faults and volcanic dykes in the Reykjanes Oblique Rift, allowing us to infer new dynamic fault processes. Conventional seismometer recordings, acquired simultaneously, validate the spectral amplitude DAS response between 0.1 and 100 Hz bandwidth. We suggest that the networks of fibre-optic telecommunication lines worldwide could be used as seismometers opening a new window for Earth hazard assessment and exploration.

## Abstract (web summary)

Imaging the internal structure of faults remains challenging using conventional seismometers. Here, the authors use fibre-optic cables used for telecommunications to obtain strain data and identify faults and volcanic dykes in Iceland and suggest that fibre-optic cables could be used for hazard assessment.

## Introduction

Seismic and ground-motion datasets quality (spatial density, accuracy, bandwidth, etc.) determines our ability to characterize crustal media properties distribution, seismic source processes and wave propagation mechanisms. These are mandatory for acute natural hazard assessment^1–3^, for efficient resource exploration^4^, and for structural health monitoring and security^5^. For example, faults are known to display different elastic properties^6^, due to the existence of a damage zone^7^. The structure and physical properties of faults control processes of dynamic rupture and/or creep. Prior to rupture, tectonic stress accumulates and rock damage grows, inducing deformation of the Earth crust^8^. In addition, dynamic stress from remote earthquakes has also been proposed as a mechanism to trigger volcanic eruptions, earthquakes, as well as micro-seismic activity^9^, and may also explain inelastic ground response in compliant fault zones^10^. Micrometre-scale deformation at faults were inferred from GPS and broadband seismological observations during the Barðabunga volcanic eruption in Iceland^11^. However, imaging the internal structure of faults with high resolution as well as inferring creeping processes of faults at sub-micrometre steps remains challenging^3^. This prevents an accurate assessment of associated near fault hazard^7^.

In order to refine images of the structure and better understand fault rupture and processes, seismology requires dense spatial coverage of individual sensors^12–14^, more accurate recording instruments and new techniques for processing data^11^. Seismic networks deployed for several decades^15^ produce waveforms with increasing quality and broader frequency content. Data from those networks fostered many discoveries and advanced knowledge, e.g. on crustal anisotropy, the core-mantle boundary, and detailed images of the crust^16^. The deployment of such networks requires great effort and resources, especially in areas where access is limited. The rising cost of maintenance makes it arduous to expand those networks much further. Alternative solutions from space are being developed^17^ but remain inaccurate. Complementary solutions on the ground have also been proposed such as including GPS measurements to detect earthquake surface wave’s characteristics^18^. So far, conventional recording instruments used in those networks provide high quality waveforms but spatially aliased data. Accurate wave-fields in space are frequently acquired by increasing the density of instruments at the surface, such as network deployment of cheap geophones^19^. Those studies address mainly local structures (typically several km^2^) and use limited frequency band (>4 Hz).

Fibre-optic technologies have been offering a range of solutions for an increasing array of applications^20^. Two sensing strategies are commonly proposed. The first strategy designs high quality sensors (higher bandwidth, resolution, etc.), which however still remain single points of observations in space^21^, whereas the seismic wave-field is constantly varying with location and time. The second strategy, referred to as distributed fibre-optic sensing, uses the entire length of an optical fibre as a sensing element allowing a marked densification of spatial sampling down to the metre scale over a distance of tens of kilometres. A passing seismic wave disturbs the sub-surface, locally stretching and compressing the ground; a buried fibre-optic cable is therefore stretched and compressed as well. Fibre-optic sensors measure the response of the optical fibre to the external forces applied to it. This can be done in a variety of ways^22^, but in general the principle involves sending a pulsed coherent optical laser signal^23^ propagating along the fibre and measuring the naturally backscattered light. The time-of-flight of the laser signal and its backscattered component are recorded and converted into a distance value using the speed of light and refractive index of the fibre. The phase of Rayleigh backscattered light along an optical fibre is well suited for monitoring dynamic strain changes, with a high spatial and high temporal resolution (Methods: Distributed fibre-optic sensing). Whereas the physical principle^24^ is named phase-OTDR (optical time domain reflectometry), its application for ground-motion detection is often referred to as distributed acoustic sensing (DAS)^25^ or distributed vibration sensing (DVS)^26^. Sensitivities down to the nano-strain are achieved with current technologies^27^.

The DAS/DVS technologies have been mainly developed in the oil and gas industry. A common application is in seismic acquisition, with active sources. Fibre-optic cables that have been previously deployed in a borehole, for communication with a downhole gauge, for example are regularly used. However, it is also possible to design and deploy dedicated cables with improved characteristics for certain applications^28^. The deployment of optical cables in boreholes allows structural underground investigation and monitoring of reservoirs properties^29^. The DAS/DVS technologies tend to complement classical vertical seismic profile (VSP) measurements^30,31^. Field studies on VSP data show that the frequency spectrum recorded with DAS/DVS is comparable to conventional geophone data^32^, where the bandwidth of the seismic record is limited by the minimum frequency generated by the source, e.g. >5–10 Hz. However, measurements performed with DAS/DVS in the laboratory^33^ produced acoustic bandwidth from 0.008 Hz to 49.5 kHz suggesting possible applications at longer seismic periods (<1 Hz) in the field^34–40^. In the following, we refer to “DAS” for simplicity.

In this study, we find new structural and dynamic features of normal faults zones within the oblique rift of Reykjanes mid-oceanic ridge. We achieve those findings by using an existing ~15-km long conventional fibre-optic cable, utilized for telecommunication in Reykjanes Peninsula (SW-Iceland). This cable was deployed in 1994 with a plough in a <1 m deep trench and covered with sandy soil and gravel. We analyse the continuous strain-rate data recorded with a dedicated acquisition system (iDAS) over 9 days in March 2015 with high sampling both in time (1000 Hz) and space (4 m). We thus demonstrate that conventional fibre-optic cables already deployed in the ground for telecommunication purposes can be used as quasi-continuous lines of highly sensitive sensors, providing spatially un-aliased strain data over a broad frequency band useful for seismological research. This spatially dense acquisition over a large distance allows (1) to record data yielding improved earthquake identification and localizations, and (2) to detect small features of the sub-surface which can then be compared to the local geology, e.g. fault zones and volcanic dykes associated to the rift.

## Results

### Validation of the DAS records

**Fig. 1:**
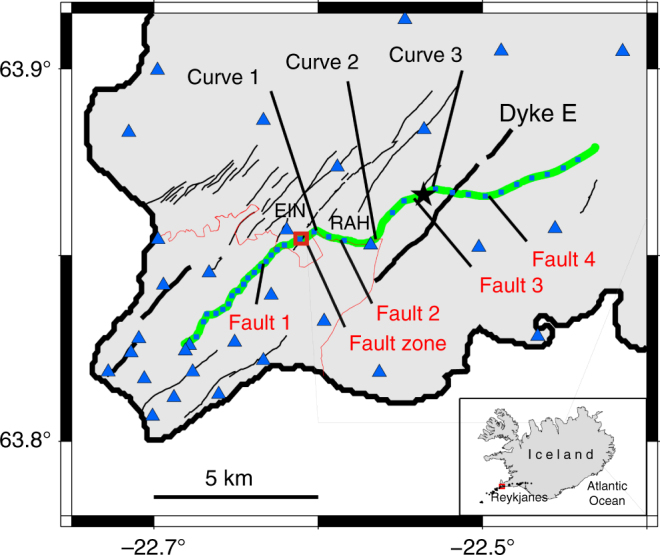
Location of the fibre-optic cable in Reykjanes and main geological features^70^. Location of the fibre-optic cable (continuous green line) from the telecommunication network (Míla Company) used for our measurements within the Reykjanes fissure swarm (black lines). Small light blue squares along the fibre-optic cable represent geophones. Blue triangles indicate broadband seismological stations from the European Project IMAGE (Integrated Methods for Advanced Geothermal Exploration) network^41,44^. RAH and EIN are the closest broadband stations to the optical cable. The thick black lines indicate a series of cones and postglacial craters, from the latest eruptive episode in Reykjanes in 1210–1240 (e.g. Dyke E = Eldvörp crater row). The black star indicates a local earthquake epicentre (depth ~3.5 km). The thin red curve indicates the limit of the Sh = Sandfellshæð lava shield (most recent lava flow), hiding most of the faults at the surface of the tip of the Peninsula. The inset represents the location of the area in Iceland (North Atlantic), with black dots being epicentres of 68 earthquakes (Supplementary Table 1) recorded during the 9 days of our optical DAS records

We process our dense ground-motion DAS strain-rate records both in terms of amplitude and frequency content to evaluate the response characteristics of the previously deployed fibre-optic cable (e.g. the ability to adequately record broadband ground-motion signals for seismological research), and to derive information about the crustal structure and rifting processes at Reykjanes Peninsula (Supplementary Note 1 and Fig. 1).

**Fig. 2:**
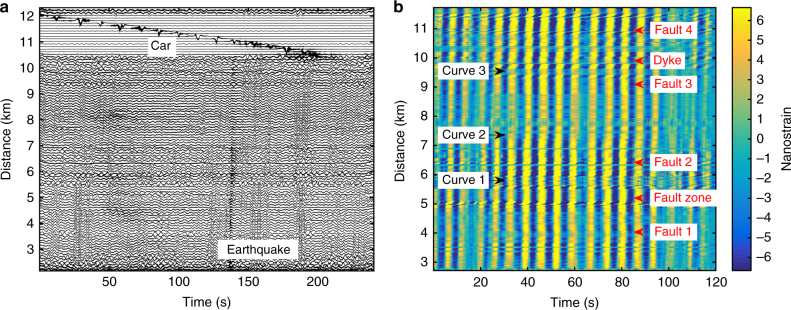
DAS records. **a** 4 min of strain signal (17 March 2015, 12:33–12:37). Only selected normalized traces (one trace out of 25, i.e. one trace every 100 m, frequency range 0.01–100 Hz) are shown. A local earthquake is revealed by higher frequencies in the signal from 135 to 140 s. Coherent oscillations of 5–6 s period correspond to ocean-driven micro-seism. Traces between 10.5 and 11.6 km with large amplitude signals correspond to a car travelling on the road along the cable (Methods: Shallow sub-surface crustal properties determination). **b** 2 min of strain record (19.03.2015, 15:27 UTC) showing micro-seism (4–6 s period) propagating from the south coast northwards along the cable. Beamforming computation (from the DAS record) indicates a source in the Atlantic Ocean, SW of Iceland. Changes in cable direction along the road (black labels) induce a change in the incidence angle of the micro-seism waves, and therefore amplitude change. Amplitudes and phases are disturbed at specific locations (indicated by the red labels), which correspond to geological features such as faults or volcanic dykes (Fig. 1)

**Fig. 3:**
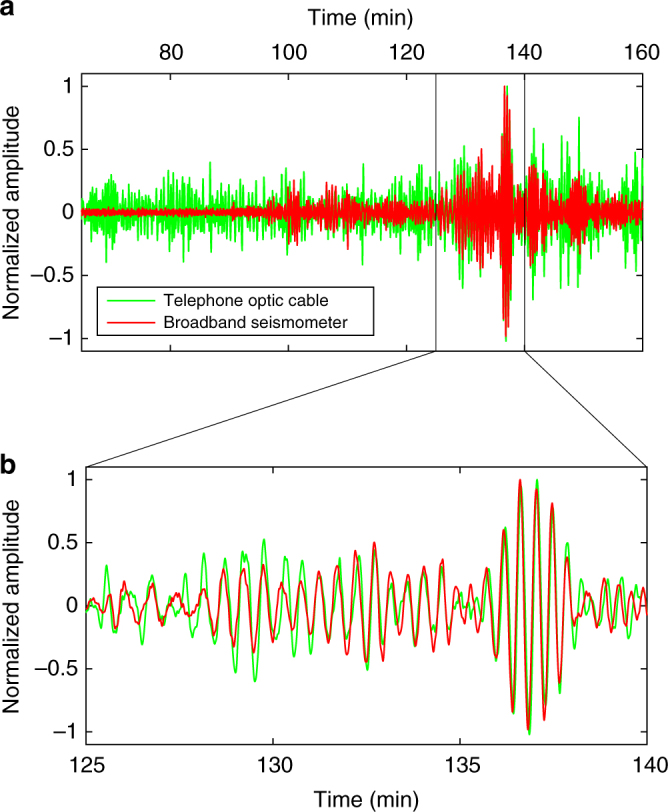
Record of a teleseism earthquake with DAS. **a** Normalized strain (green curve) recorded during an Mb ~6.2 (USGS) earthquake (Kota Ternate, Indonesia, 2015-03-17 22:12:28 UTC, 1.669°N; 126.522°E, 44 km depth) superimposed with the normalized velocity record (red curve) from the broadband station RAH (80 m from the optical cable). Data are filtered between 16 and 50 s corresponding to highest amplitudes for surface waves of remote earthquakes. **b** Zoom from **a** showing good phase correspondence between seismometer velocity record and DAS strain records at 20 s period

**Fig. 4:**
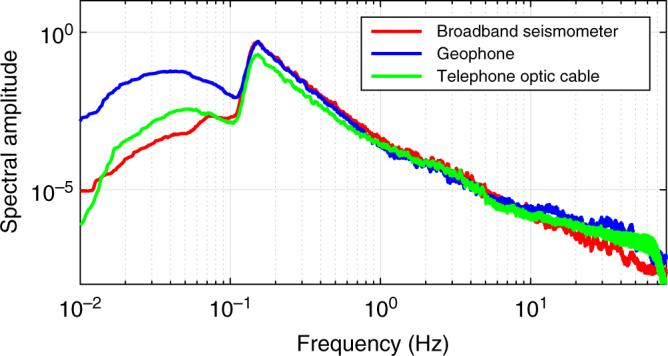
DAS record conversion to seismic data. True amplitude spectra of displacements of 1 h (20.03.2015 5:00-06:00) noise record for DAS (green), geophone (natural frequency of 4.5 Hz, blue) and broadband (flat amplitude response between 0.008 and 100 Hz, red) records, respectively, after instrumental corrections (Methods: Instrumental correction of records from the iDAS system)

We obtain characteristics of the DAS strain signals as recorded along the profile defined by the fibre cable for ground-motion studies. Over a broad frequency band, we show that recorded strain rate signals are meaningful: after we corrected for the instrumental response of both DAS and seismometer data, the DAS signals accurately match those derived from the seismometers (in terms of the frequency content as well as the phase characteristics). This suggests the data acquired by the DAS is a valid representation of the near surface ground deformation, and can therefore be used for shallow crustal exploration and monitoring with similar performance as broadband seismological sensors typically deployed in an array (Figs. 2–4; Methods: Strain and displacement and velocity determination).

We identify signals generated from a large variety of both anthropogenic and natural sources (0.05 Hz up to >100 Hz). High frequency signals (1–100 Hz) are mostly generated by anthropogenic sources, such as passing cars, fluid circulating in pipes of nearby geothermal power stations, hammer shots on the ground, people walking, and distant active explosion shots (Figs. 2–4). In addition, we detected local earthquakes (0.5–20 Hz) associated with the seismic activity of the Mid-Atlantic Ridge (Supplementary Table 1). We observed oceanic micro-seism (ambient seismic noise with signal frequencies from 0.1 to several Hz), as well as Rayleigh waves from large remote earthquakes (with ~20 s signal period, Fig. 3 and Supplementary Table 2). Figure 3 shows the filtered records (range 5–40 s) from the optic cable and broadband seismometer for a Mb ~6.2 earthquake in Indonesia, including surface waves (period ranging 10–30 s).

We validate our observations with records from co-located short-period three-component geophones and broadband seismic sensors deployed in the vicinity of the optic cable^41^ (Fig. 1; Method: Cable localisation). Waveforms from individual traces along the optical fibre exhibit high coherency, as well as with signals from broadband seismometers located along the cable (stations RAH and EIN, Fig. 1). Broadband signals (0.1–10 Hz) associated with the ground deformation due to passing cars along the fibre can be observed (Fig. 2a). We retrieve local average sub-surface ground elastic properties from the response to a car using simple ground deformation models (Methods).

To interpret DAS data, it is of primary importance to evaluate the performance of the iDAS system with respect to traditional acquisition seismic systems^22,25–27^. We compare single record of the DAS data set with records from the closest geophone and from a nearby broadband seismometer. We perform this data comparison both on ambient noise (Fig. 4) and during earthquakes. Several technical issues must be first solved. (1) We locate each DAS trace along the cable with a final spatial uncertainty of ±5 m, using hammer shots and GPS locations from the geophone data. (Fig. 1 and Methods). (2) We orient the recorded horizontal components of the seismometers along the local direction given by the cable. (3) We correct the instrumental responses of the iDAS system (Methods), of the geophones and the broadband seismometer on the respective record, prior to the determination of the seismic signal phase and amplitude.

The applied instrumental correction for the iDAS system can be used to accurately represent amplitude and phase of the seismic signal as confirmed by the comparison to classical seismic recording equipment (Fig. 4). Although DAS data can be converted to ground velocity, our conversion is valid in a certain frequency range only. Therefore, it is more convenient to follow a different approach when analysing fibre-optic DAS data, as demonstrated in the analysis and modelling of the ground deformation due to a passing car (Methods: Shallow sub-surface crustal properties determination). DAS systems typically measure strain rate or strain between two neighbouring positions within an optical fibre. The integration of strain data in space (along the cable) allows the calculation of the displacement of each data point relative to a chosen reference. Given an appropriate integration length, amplitude and phase, static local deformations and passing seismic signals can be quantified, removing the need to apply the instrumental correction. For localized deformations, the integration length must exceed the distance over which the deformation occurs. For passing seismic waves, at least half of the maximum wavelength must be integrated to properly quantify seismic amplitudes (Nyquist’s theorem). The required length for long period signals easily exceeds the length of the sensor system (cable), which is typically a couple of km. While focusing on single traces and applying the instrumental correction is appropriate to analyse passing waves with periods from 0.01 s to several minutes, high frequency signals as well as localized (static) deformation can be accurately analysed by integrating DAS data in space. In the following, we apply time-integration of the strain rate to obtain strain and space-integration to obtain displacement (Methods: "Strain and displacement and velocity determination").

### Earthquake identification and localisation

Accurate earthquake localisation is still one of the challenges in seismology^42^. The accuracy of the seismic wave velocity model and the network design determine the earthquake hypocentre accuracy. The crustal structure of Reykjanes was the topic of investigation in several geophysical and particularly seismic studies^43^. The EC funded project “IMAGE” performed new passive seismic data acquisition, including deployment of Ocean Bottom Seismometers^44^. A structural analysis was performed using classical and modern seismic methods^41,45,46^. In particular, we jointly inverted earthquakes locations and P-wave (*V*_p_) and/or *V*_p_/*V*_s_ ratio models of Reykjanes by a local travel time tomography^41^. From surface down to 4–5 km, seismic velocity increases rapidly from 1.8 to 4.2 km s^−1^, which is a typical velocity gradient for oceanic crust. *V*_p_/*V*_s_ ratios are indicative of the absence of large magma reservoirs in Reykjanes, which is also confirmed by the recent IDDP-2 drilling^47^.

**Fig. 5:**
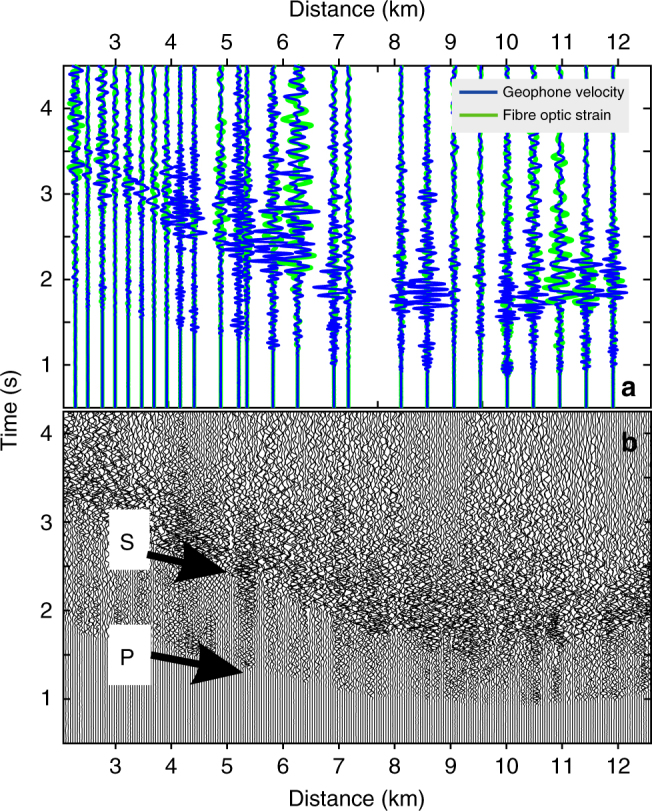
Records of a local earthquake. **a** Geophone record (blue) of an Ml ~1.2 local earthquake (23.03.2015, 16:07:08.5 U.T.C.—Iceland Meteorological Office) and fibre-optic (green) record at the corresponding locations of the geophone. **b** DAS record of the same earthquake as in **a**

**Fig. 6:**
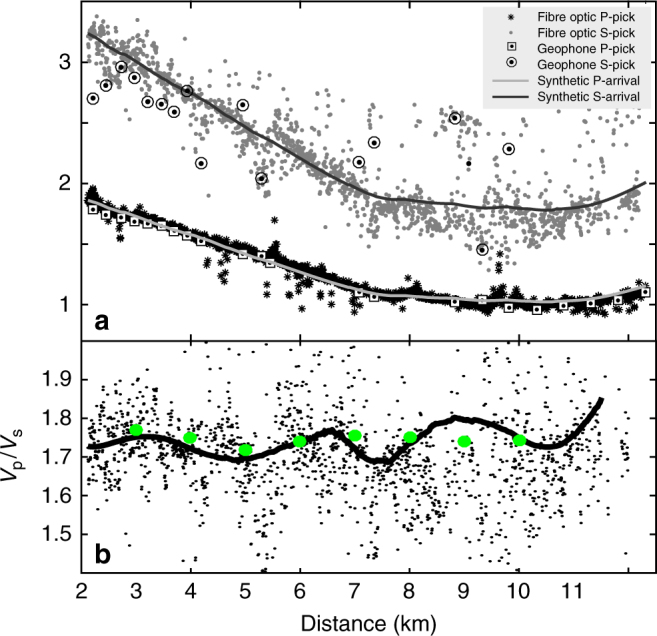
Exploration studies using conventional seismological methods and a fibre-optic telecommunication line. **a** P- and S-waves’ travel times automatically picked along the profile: each symbol represents a P- (black star) and S- (grey dots) arrival times on the DAS records. The white squares with black dot and the white circle with black dot correspond to P- and S-wave travel times, respectively, picked on the geophone records with the same automatic picker. The continuous grey (black) lines correspond to theoretical arrival times for the inverted hypocentre using P- and S-wave picks from the cable (respectively). **b** Observed *V*_p_/*V*_s_ ratio computed at all traces and compared with the results obtained from the travel time tomography (green dots) obtained from more than 2000 local earthquakes over 1.5 years^41^. The black line corresponds to the polynomial (Savitsky–Golay) smoothing filter of order 5 with size frame ~3 km long through the fibre-optic *V*_p_/*V*_s_ individual values

We focus here on one particular earthquake (Ml ~1.2) that occurred almost beneath the cable at 3 ± 1 km depth in the tomographic model^41^. Figure 5 shows the geophone and DAS records along the cable for this small local earthquake. We use an automatic picker based on Akaike Information Criteria to obtain more than 500 valid P- and S-wave arrival times along the cable. P-wave picks have good coherency between neighbouring traces whereas S-wave have poorer coherency (Fig. 6). Although the telecommunication cable geometry was for sure not designed for earthquake monitoring, we obtain a hypocentre location using P- and S-wave travel times automatically picked on all traces recorded along the cable. We find a hypocentre similar to the one obtained from conventional seismological network^41^. The probability density function (pdf) of the earthquake hypocentre location is obtained using only the travel time data derived from the DAS record (Supplementary Figure 7). The pdf locates the hypocentre within few hundred metres from the hypocentre location obtained in the IMAGE velocity model demonstrating that the iDAS system can be used for earthquake monitoring and localization. In addition, we note the rather good match both in amplitude and trend between the derived *V*_p_/*V*_s_ ratio obtained from DAS records for this earthquake (only one) and the *V*_p_/*V*_s_ ratio from 3D local tomography (Fig. 6b).

### Crustal structural features detection

**Fig. 7:**
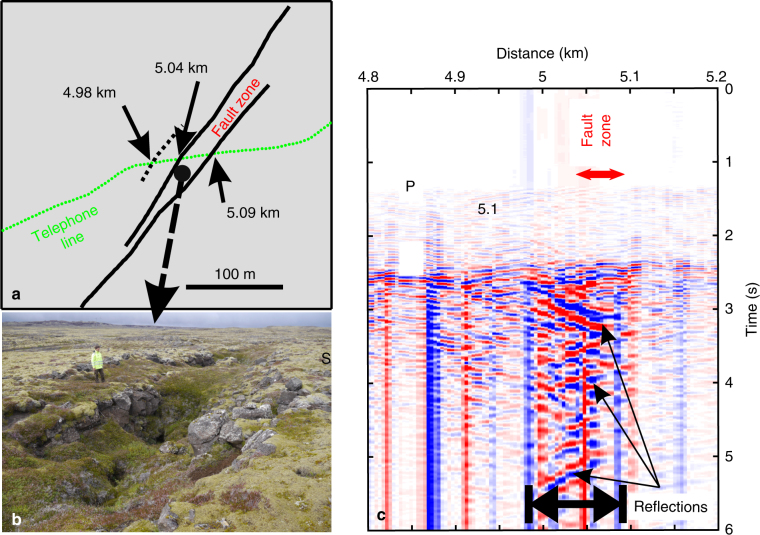
Structure of a fault damage zone within an active geological rift. **a** The road and the cable (distance ~5 km) cross several faults, e.g. a clearly visible fault zone with more loose material in the field (between 5.04 and 5.09 km). **b** The fault damage zone is visible by the ~50–60 m wide depression area (picture taken at ~100 m SW of the road, looking towards SW). Note that at the cable location no depression area is visible. The depression is only the surface expression at the position of the picture (Picture Martin Lipus, GFZ). **c** Short record (6 s) of strain phases from a local earthquake (Fig. 5) trapped in the fault damage zone. Phases are reflected until ~4.98 km, which may indicate a hidden fault with surface expression. Waves inside and outside the fault zone have different apparent velocities

Geometrical and physical sub-surface properties of the damage fault zone could be derived using waves from local earthquakes trapped within a fault damage zone (Fig. 7). From the geological and structural maps of Reykjanes^48^, we notice that the fibre-optic cable crosses several tectonic and volcanic features, i.e. volcanic dykes and faults. One prominent fault zone crosses the cable at a distance of about 5 km along the road (Fig. 1). This fault zone crops out along the rift to the north and the south of the road over several kilometres. The Eldvörp crater row crosses the cable at a distance of about 10.5 km. Several observations indicate the signature of those geological features in the DAS record. For example, in Figs. 5b and 6a note the larger delay of P-wave arrival time at distance ~5 km, corresponding to the presence of a fault zone. Similarly, note the faster arrival times at distance ~10.5 km, where the cable crosses the Eldvörp crater row. In Fig. 5d, *V*_p_/*V*_s_ ratio along the cable shows kinks located at faults. Those features cannot be seen in the sparser geophone records.

Dense arrays of seismometers give the opportunity to better image the sub-surface, especially using recently developed imaging techniques, such as ambient noise cross-correlation and auto-correlation technologies^49^. Even when classical sources (earthquakes, etc.) are absent, we show that ambient noise analysis techniques reveal wave disturbances associated with fault zones (Method: Ambient noise interferometric techniques with DAS records). As ambient noise is rather strong in Reykjanes^45^, we computed autocorrelations and cross-correlations in order to illustrate the detection and imaging capabilities of those methods with DAS records (Supplementary Figure 8). We observe similar quality and good coherency between autocorrelations from DAS record and co-located geophones and the classical X shape of wave propagation from the virtual source towards larger offsets. Those results demonstrate that geophysical studies (detection, mapping, localisation, monitoring, etc.) from correlations methods could be performed using available telecommunication cable networks for further structural interpretation.

### Towards imaging the damage zone within fault systems

From the analysis of trapped waves in fault damage zones, P-wave and S-wave velocities were found to be typically 35 to 45% lower than those of the surroundings rocks in California^7^. For all earthquakes recorded by the iDAS system, we observed similar characteristic wave-field features at several places along the telecommunication cable. As an example, we focus on a fault damage zone (FDZ) with a clear surface expression (Fig. 7). We observe an increase in both, duration and amplitude of trapped waves excited by local earthquakes. Interestingly, we observe similar trapped-wave features in the micro-seismic noise, even when local earthquakes are absent (Supplementary Figure 9). Due to the existence of the fault zone with altered properties (decreased velocities), we observe a phase shift and increased amplitude of micro-seism in the fault damage zone. This observation suggests that a local structural feature associated to the fault zone is responsible for the increased amplitude and longer codas. Such trapped phases are often seen, although not normally with this high spatial sampling. Thus, the spatially dense fibre-optic cable records allow us to follow details of the earthquake phase’s propagation within the fault zone. To our knowledge, it is the first time that such details of the wave propagation are observed within a fault damage zone. From the geological observation in the field (Fig. 1), the fault zone appears to have a width of about 60 ± 10 m at the cable location. This is in agreement with the distance over which we see larger amplitudes of the seismic signals and where we observe seismic phases bouncing from one side to the other side of the fault zone (Fig. 7c). From the slope of the phases propagating within the fault damage zone, we could estimate an apparent velocity of about 300-400 m s^−1^, which correspond to a velocity slower by 30-40% than outside the fault damage zone. However, we also observe reflected phases further south-west of the fault zone visible at the surface, until 4.98 km. This observation suggests that we may have discovered a hidden fault, which could mark the limit of the fault damage zone, as indicated by the trapped waves. As there are many faults in rift zones, and as observed in our records (Figs. 2b, 5 and 6), this result may support the recent suggested statement that crust in a geological rifting environment is weaker than elsewhere^11^.

### Fault dynamic processes triggered by remote earthquakes

**Fig. 8:**
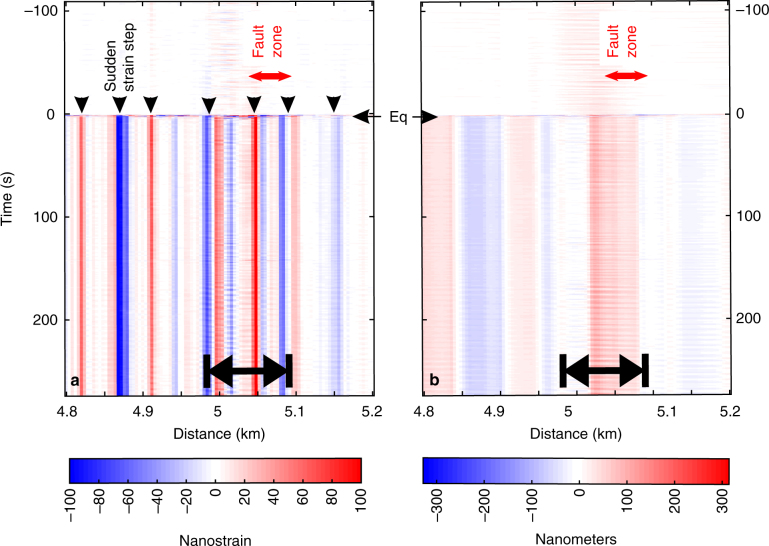
Dynamics of a fault damage zone within an active geological rift. **a** Extensive record (400 s) of strain observed in the vicinity of the damage fault zone, from 100 s before, during and 300 s after the earthquake (Figs. 6 and 7). Sudden strain steps (black arrows indicated at the time 0) occur over several neighbouring traces simultaneously to the waves of the earthquake. Strain remains with the same value for at least 300 s, possibly more. The location of the steps correspond to locations of geological features observed in the field, e.g. faults. **b** Displacement computed by spatial integration at selected traces along the same section of the cable as in Fig. 7a and c. The displacement is directly obtained from the spatial integration of the strain in **a** over a 60-m-long sliding window. Eq: earthquake

Our DAS data analysis brings new insights on geological rifting processes. By analysing the ground position before and after local earthquakes we find relative quasi-static displacement offsets that do not relax within a period of a few minutes. This is seen in the faults (Fig. 8) which are crossed by the optical fibre line (Fig. 1). In Fig. 7c, records with much larger positive and negative strain amplitudes suddenly appear at specific locations when the earthquake waves are passing by. The strain in the fibre remains after the seismic waves dissipate (Fig. 8a). Structural features such as the low wave velocities of the damaged zone within the fault may contribute to explain such observations. However, these sudden local strain jumps are not observed for all earthquakes, but always appear at the same locations. The location of the jumps correspond to locations of geological features observed in the field, e.g. faults. To better understand processes generating those records, we calculated displacement records by spatial integration of strain over a few traces (typically over 40–60 m). Wave-fields generated by remote (or local) earthquakes may or may not trigger sub-micrometre static displacement shifts of the ground close to weaker crustal features, such as a fault zone.

## Discussion

Development and research to evaluate and expand the capabilities of fibre-optic cables^28,50^ are required, so that newly deployed cables could fulfill the requirements for exploration and monitoring (e.g. separating the fibre sensitivity into the three components). However, as demonstrated here, existing buried optical fibre telecommunication infrastructure offers a cheap alternative to deployment of dense networks of various sensors and new optical cables. We emphasize that our results are obtained with a conventional fibre-optic cable within a telecommunication network, not been specifically designed for seismic purposes. This points to the extraordinary potential of this technology for new applications in Earth hazard assessment and exploration all over the world.

We have demonstrated that the DAS technology using fibre-optic cables from existing telecommunication networks has many applications. We analysed the records with metre-scale resolution over a broad frequency band (tens of seconds until tens of Hz), for sub-surface m- to km-scale exploration. We showed that the DAS records can be compared with conventional seismic records. We report applications focussing on sub-surface exploration for elastic rock properties and earthquake monitoring. We also discovered unusual wave-field features of fault structures and dynamics in a geological active rift (Reykjanes, Iceland). The spatial density increase over a long distance is one of the major advantages for obtaining detailed information on Earth property distribution. With only one earthquake (we did not use other earthquakes), we determined structural rock properties at the km scale and at the fault damage zone scale and inferred hints on strain accumulation and creeping processes. For instance, the trapped seismic phases observed in a fault damage zone are single- or multiple- reflected phases at two reflectors or more, which we interpret as being fault zone boundaries, defining the fault damage zone, as inferred from our seismic observations. Reflected waves in the micro-seism suggest that seismic energy is trapped in the fault zone at various frequencies. Those results suggest that when applied to many earthquakes, even more detailed information could be retrieved. Our observations also reveal potential creeping processes at faults and fault damage zones induced by impinging seismic waves from local earthquakes. Dynamic strain perturbations due to the passing waves from local earthquakes trigger relative displacements that may correspond to tiny aseismic fault movements, interpreted as creeping processes. Micro-seism may influence fault creeping processes as well as remote earthquakes. Further analysis in this direction could help solve questions related to co-seismic fault deformation^3,10,51^, understand fault preparation prior to large earthquakes as well as aseismic deformation. Those results open a new window for the study of remote triggering of earthquakes and stress build-up at faults, especially in cities where fibre-optic cable networks may be dense and where seismic hazard is high (San Francisco, Mexico, Tokyo, etc).

The dramatic increase in sensor density over a large distance with unprecedented acquisition characteristics (sampling in space and time and over a large frequency band) suggests that scientists could test new approaches and unconventional data processing, which then might obtain more accurate results compared to classical seismological methods. The DAS technology thus offers a great potential for Earth exploration and natural hazard assessment, offering new scientific research opportunities. Improving the sensitivity of cables in existing networks, determining accurate position and orientation of the observed traces and understanding details of the ground/cable coupling issues^25^ are certainly great challenges when exploiting buried optical communication lines. For the situation on Reykjanes Peninsula, the analysis of the stress transfer from the ground to the sensing core of the fibre is more than 90% efficient for seismic frequencies between several 10’s Hz and long seismic periods^52^. By demonstrating that the data acquired on a telecommunication network of fibre-optic cable fulfills many requirements for improvement of seismological analysis, we foresee a vibrant future for the use of optical sensor technologies in seismology applications. Besides the deployment of new dedicated and improved cables in order to allow for observation of the full strain tensor, existing infrastructures may allow for simultaneous monitoring of strain and ground motion for natural hazard assessment. They could help in more accurate earthquake localisation and focal mechanism determination, volcanic activity monitoring and a more complete characterisation of the range of volcanic and seismic sources, seismic hazard assessment, global seismology studies, exploration, etc.

We also suggest that our results may open the door to new ways of data processing^53^. With the advent of spatially un-aliased, i.e. densely sampled seismic data, array analysis methods (e.g. Helmholtz tomography) becomes easier to implement^54^, potentially providing a huge improvement in resolution by directly inverting and/or imaging sub-surface structures utilizing full wave-field recordings.

Can we also envisage a change of paradigm in theoretical seismology? The classical stress/displacement approach in seismology uses the basics of mechanics and observational seismology is based mostly on displacement and/or velocity and/or acceleration sensor recordings. With a fibre-optic cable providing equivalent broadband seismometers records, the gradient of the displacement, i.e. the strain, is uniquely measured at many more locations than before. We believe new processing methods may be needed. New mathematical progress for tomography has been recently discovered^55^ but their application is hindered by the lack of information at the Earth surface^56^. Since fibre-optic lines are deployed very widely and densely on Earth, e.g. for telecommunication (~10^6^ km cable deployed under the sea^57^), we anticipate that our results will open a new era for strain and ground-motion acquisition at all scales, for both seismic processing and modelling. For instance and non-exclusively, monitoring of underground explosions in the framework of the CTBTO, volcano monitoring, seismic hazard assessment, landslide monitoring and, global seismology using transatlantic optical cables could benefit from this technology with current and future infrastructures. We may also envisage dedicated experiments to compare new instrument development in rotational seismology^58^, and detailed studies of surface wave properties^59^. Many other applications, like car traffic monitoring, theft protection, city underground monitoring^37^ will promote telecommunication companies as actors for Earth hazard monitoring, exploration and security enhancement for the benefit of research^60^ and human societies.

## Methods

### Distributed fibre-optic strain sensing

Various optical architectures have been used to interrogate the backscattered Rayleigh light, ranging from relatively simple, coherent-OTDR (optical time domain reflectometer) schemes, which are unable to determine acoustic phase and so are unsuitable for seismic measurements, to more complex arrangements which provide the full acoustic amplitude, frequency and phase. Both the simple and complex range of systems are generally described as DAS^25,33^ or DVS^26^, though only the phase sensitive variants have been successfully used for seismic applications^60^. The description of the underlying sensing principles of the DAS/DVS technology has been reported^22,61,62^. When a laser pulse is launched into an optical fibre, a fraction of the light is elastically scattered (Rayleigh scattering) due to random inhomogeneity distribution in the glass fibre material. During interrogation of an optical fibre, the backscattered photons can be detected. The position of the scattering inhomogeneity within the fibre can be calculated based on the speed of light within the fibre. This method is called optical time domain reflectometry (OTDR)^63^. If a coherent laser pulse is launched into the fibre, with appropriate optical processing, not only the amplitude but also the phase of the backscattered photons can be analysed (phase-OTDR). For any section of the fibre, the phase-difference ∆*ϕ* of photons scattered at both ends of that section is linearly related to the length of this section^27^. When the section of the sensing fibre is unperturbed, the length and, consequently, the phase-difference ∆*ϕ* remains unchanged. Any perturbation inducing a strain *ε* on the fibre will change that difference. The strain rate can therefore be mapped along the sensing fibre by examining the changes in the phase of the elastically backscattered photons between successive measurements. For example, an imbalanced Mach-Zehnder interferometer has been used to measure dynamic strain changes along the fibre^64^. A fibre-optic cable can hence be considered a system consisting of a large number of one component relative strain gauges. State of the art DAS systems are capable of quantifying the frequency, amplitude, phase and location of dynamic perturbations anywhere along the sensing fibre. Measurement systems with the capability to resolve perturbations of 40 nϵ are reported^27^.

### Strain and displacement and velocity determination

In our study, we define the DAS system as comprising the deployed fibre-optic cable (sensor) and the iDAS interrogation system^33^. The phase-difference is a measure of the relative travel time and hence the relative distance. The physical distance over which the phase-difference measurement is performed, is referred to as the “gauge length” d*x*^26,61^. Comparing successive pulses, phase changes are directly related to the distance changes and therefore the strain rate in direction of the fibre can be recorded. In the iDAS system, each digital sample is indexed by the centre location of a moving window along a cable’s fibre core (the sample’s ‘channel’, *x*) and recording time (the sample’s ‘time’, *t*). Thus, if *u(x,t)* represents the dynamic displacement of the fibre, the DAS observation DASobs at axial location *x* and time *t* is a measure of the strain rate at the distance *x* from the iDAS recorder and is expressed by:

1 $${\mathrm{DAS}}\,{\mathrm{obs}}(x,t) = 	\left[ {u\left( {x + \frac{{{\mathrm{d}}x}}{2},t} \right) - u\left( {x - \frac{{{\mathrm{d}}x}}{2},t} \right)} \right] \\ 	- \left[ {u\left( {x + \frac{{{\mathrm{d}}x}}{2},t - {\mathrm{d}}t} \right) - u\left( {x - \frac{{{\mathrm{d}}x}}{2},t - {\mathrm{d}}t} \right)} \right]$$

where d*x* and d*t* are the spatial gauge length and temporal sample interval, respectively. The typical gauge length for seismic applications spans d*x* = ~10 m. The longer the gauge length, the more sensitive the DAS system with increased signal to noise ratio. Wavelength *λ* below d*x*/2 can however not be resolved (Nyquist’s theorem in space^26^). Together with the spatial sampling, the gauge length determines the dependency between individual seismic traces. Although data can be acquired with a high spatial resolution, the assumption of independent traces only holds true if the gauge length of neighbouring traces does not overlap. In order to achieve a sufficient intensity of the backscattered light for each data point and every laser pulse, the laser pulse has a given width and the backscattered light has to be integrated for a given time. Both, the pulse width as well as the integration time acts as a moving averaging filtering in space^26^. In some implementations, post-acquisition averaging of individual traces is applied to suppress unwanted optical noise and an increase in seismic signal/noise ratio.

DAS data can be equivalently regarded either as an estimate of the fibre strain-rate

2 $$\frac{\partial }{{\partial t}}\left( {\frac{{\partial u}}{{\partial x}}} \right)$$

or as an estimate of the spatial derivative of fibre particle velocity

3 $$\frac{\partial }{{\partial x}}\left( {\frac{{\partial u}}{{\partial t}}} \right)$$

If we integrate the distributed strain-rate along the optical fibre with respect to time, local strain can be estimated for every section along the optical fibre. If we integrate the local strain with respect to space, the relative displacement can be calculated at all points along the profile. Note that by differentiating with respect to time, we obtain an estimate of the velocity of the ground, which we may compare to local broadband seismometer records. We compare spectral data in displacements (Fig. 4).

### Cable localisation

Using an optical cable at the surface is driven by the idea that there could potentially be a larger range of applications both in hazard assessment and crustal exploration, although applications at the surface are described to be more challenging^26^. Instead of deploying a new dedicated cable (with great amount of expenses), we use an optical fibre within the commercial telecommunication network on the Reykjanes peninsula, SW-Iceland. The geographical position of the cable was provided by the telecommunication provider (Mila). The optical data is given in terms of distance along the optical fibre within the cable to the iDAS recorder. Each trace of the DAS record has an associated distance from the iDAS recorder. In order to locate and check the accuracy of the observed distances from the DAS record, we deployed an array of 33 geophones every 250–500 m along the cable. We determine the position of each geophone using precise differential GPS (accuracy better than ~0.5 m). To locate individual DAS traces, we assign the geographical position of the geophones to the closest optical trace. At each geophone, we performed six successive hammer shots for calibration purpose. We compare DAS records of hammer shots in the vicinity of each geophone with the records of these shots at different traces along the cable. We identify the DAS trace with the earliest arrival time of seismic waves associated to the hammer shots. For every geophone position, the shortest distance to the position of the cable was calculated and the geographical position of this point assigned to the identified DAS trace. In between individual reference points, the geographical position was linearly interpolated along the cable. To verify the positions of the traces in the intervals between geophones, the distance between individual DAS traces is calculated using the number of traces and the distance along the cable and given by the geophone localisation. We are able to localize every shot with an accuracy of the sampling resolution along the optical fibre (i.e. 4 m). The localization accuracy for records located in the range over which geophones were deployed, is therefore in the order of 10 m for distant traces to the next shot point. The sensitivity of a linear fibre is decreasing with increasing angle of the incident wave with respect to the cable direction. Therefore, the comparison of the DAS data with other type of records (geophone and broadband seismometer) requires the projection of seismic motions (displacement, velocity or acceleration) in the local direction of the cable. We oriented the broadband sensors using an Octans (IXSEA) gyro-compass^65^.

### Shallow sub-surface crustal properties determination

The iDAS system is able to record the ground deformation associated to cars passing by along the cable (Fig. 2a and Supplementary Fig. 6). We use here those records to locally determine average rock properties beneath the road. To predict the deformation of the ground to the car’s weight, we model the car by a series of 4 point loads moving on an isotropic semi-infinite elastic half-space^66^. As the speed of the car is slow compared to the Rayleigh wave velocity in the ground, we use the Flamant–Boussinesq approximation theory describing the static deformation of a point load on the ground surface^67^. In this theory, the ground displacement *u*(*u*_*x*_,*u*_*y*,_*u*_*z*_) in the *i*th direction is given by

4 $$u_i = \frac{F}{{4\pi {\mathrm{\mu }}}}\left[ {\frac{{x_3x_i}}{{r^3}} + \left( {3 - 4\upsilon } \right)\frac{{\delta _{i3}}}{r} - \frac{{\left( {1 - 2\upsilon } \right)}}{{r + x_3}}\left( {\delta _{3i} + \frac{{x_i}}{r}} \right)} \right]$$

where *F* is the force of the point load (weight of the car), *μ* the shear modulus of the rock, $\upsilon$ the Poisson ratio, $r = \sqrt {u_x^2 + u_y^2 + u_z^2}$, and *δ* is the Kronecker sign (Einstein notation). The centre of mass of the car (we computed 4 mass contributions at the locations of the 4 wheels) is assumed to be located at a constant distance of 2.5 m from the cable. The shape of the strain trace with time at one location is dependent on the speed and weight of the car, on the distance to the cable, and on the elastic properties of the ground, e.g. P-wave velocity. Supplementary Fig. 6 shows an example of ground property determination with this approach at one location along the cable. The best match of the deformation curve we found for a car moving at ~25 km h^−1^ with a sub-surface P-wave velocity of ~750 m s^−1^. Note the almost perfect match between observations and our simple prediction. The P-wave velocity is consistent with velocities obtained (~500–1000 m s^−^^1^) from the refraction analysis of the seismic waves generated by hammer test shots^68^. We could use this method to derive a profile of sub-surface velocities long the cable, a task that we will pursue in a further study.

### Instrumental correction of records from the iDAS system

The iDAS system measures strain rate (Methods: Strain and displacement and velocity determination). In order to calibrate the amplitude and phase responses of the recorded signal by the iDAS system, an impulse displacement signal is sent in the cable and the output strain is measured at different frequencies. The amplitude and phase responses both depend on the frequency (Supplementary Figure 1). To retrieve the true amplitude over a frequency band of interest (<100 Hz) for our seismic applications, we should correct the recorded signal from the amplitude and phase shift introduced by the recording unit (iDAS). We focus only on the frequency of interest for our seismological applications: from <0.01 Hz to 100 Hz. In this frequency band, the response can be modelled with simple functions. We first deal with the dependency of the instrumental wave response with an apparent ground velocity *v*. The response of the iDAS is better expressed in apparent wavelength *λ* along the cable. As *v* = *λf*, *f* being the frequency, we can express the response as a non-velocity dependent function with the wavelength (Supplementary Fig. 2): the gain and phase responses are the same for all wave velocities. We also notice that the amplitude response is linear with wavelengths above roughly 20 m. The seismic frequencies of interest are about <100 Hz, therefore wavelengths of interest for us are above 15–50 m depending on the ground velocity. A simplified instrumental response can be used. We note that the amplitude decay with diminishing frequency is almost linear below 100 Hz (in logarithm versus logarithm scales). We also note that the phase is almost constant below 10–20 Hz (in semi-logarithm scale). We express both amplitude and phase responses as a linear function of the wavelength or frequency. We correct from the recorded signals for the iDAS instrumental response by multiplying, in the Fourier domain, the amplitude by the simple function C*λ, *C* being a constant. If we apply the correction to the instrumental response, we can calibrate this constant to *C* = 0.0159, by imposing the corrected instrumental response to amplitude 1 for the frequencies of interest, as shown in Supplementary Fig. 3. We also note that the phase shift is constant *π*/2 for long wavelengths (above 100 m). In practice, the processing steps to perform the restitution of the “true” ground motion, consists of integrating strain rate into strain, as iDAS records data as strain rate, and as the instrumental response is expressed in strain. We assume that the initial strain is zero all along the cable, on the basis that the average strain along the cable is zero. Then, the correction is applied in the frequency domain as shown in Supplementary Fig. 4. The restitution depends on the velocity of the medium. Supplementary Fig. 5 shows smoothed version of the restitution spectra for different velocities. Differences are due to different amplitude and phase responses of the instrumental correction at different velocities. Note that the transfer function that we used is valid only for the gauge length used (10 m).

### Probability density function of an earthquake location

The probability density function is, in inversion theory, a way to represent the difference between observations and a model. In order to find the hypocentre location that minimises the misfit difference between the synthetic travel times and DAS observations in the least square sense (RMS), we performed systematic computation of synthetic travel times for many hypothetic hypocentres within a 3D grid around the hypocentre location found by the 3D tomographic velocity model in Reykjanes. Each hypothetic hypocentres give a misfit value with respect to observations, represented in the Supplementary Fig. 7. The DAS cable observations are sufficient to define an area where the true hypocentre might be. The minimum misfit value is found at a location which is at <1 km from the hypocentre determined from the 3D travel time tomography.

### Ambient noise interferometric techniques with DAS records

We used ambient noise interferometric techniques to generate source gathers by cross-correlating DAS records at different positions (Supplementary Fig. 8). Results indicate mostly one-sided correlation because ambient noise has a strong directivity, the sources being located in the Atlantic Ocean (South-Western). Strong perturbation of ambient noise amplitude occurs at the location of fault damage zones. Other disturbances in the signal amplitude and phase (Supplementary Fig. 8) reveal various features visible in the field (such as directional changes of the cable at curves of the road), but also other features that we cannot identify accurately, without more field inspection. An example of auto-correlation computed at specific DAS/DVS records, corresponding to the location of the geophones and along the whole profile are shown in Supplementary Fig. 8b and c.

### Data availability

The fibre-optic data and the geophone datasets^69^ generated and analysed in the current study and that supports the finding of this study are accessible via the data repository of “GFZ Data Services” (10.5880/GFZ.6.2.2018.003).

## Electronic supplementary material

Supplementary Information

Peer Review Report
