# Supplementary material for: Dynamic strain determination using fibre-optic cables allows imaging of seismological and structural features
Source: Nat Commun. 2018 Jul 3;9:2509. doi: 10.1038/s41467-018-04860-y (PMC6030137; doi:10.1038/s41467-018-04860-y)
Supplement: Supplementary file 1 — Supplementary Information [file 41467_2018_4860_MOESM1_ESM.pdf]

# Dynamic strain determination using fibre-optic cables allows imaging of seismological and structural features

Jousset et al.

## Supplementary material

**Supplementary Note 1 | Summary of the geology of Reykjanes peninsula, SW Iceland**

The Reykjanes Peninsula is located at the tip of SW Iceland within the Western Volcanic Zone[1]. It is the on-shore prolongation of the Reykjanes Ridge (Northeast Atlantic Ocean). On Reykjanes, the extensional tectonic activity between the North American and Eurasian tectonic plates (2 cm/year, since 6-7 Ma) induce intense seismic activity associated with rifting processes with faults and several recent volcanic systems, with young and highly permeable basaltic formations of Pleistocene age. Tholeiitic basalts range from picrite basalts (oldest) to olivine tholeiites to tholeiites (youngest)[2]. Dike intrusions at depth provide the heat source for geothermal systems[3]. Some of those intrusions reach ground surface and produce eruptions in episodic intervals of roughly 1000 years. The most recent volcanic eruption on Reykjanes occurred as a row of scoria and spatter cones in 1210-1240 AD in the Svartsengi volcanic system (Eldvörp dyke). The Eldvörp crater row extends from the south coast about 10 km inland (Fig. 1). The lava emitted from the crater row is one of the most voluminous Holocene lava flows on the Reykjanes Peninsula, covering c. 20 $km^2$. Faults on Reykjanes are numerous and mostly exhibit normal mode, with sinistral shear components[4] due to the oblique character of the ridge compared to its opening in this area. Faults are partly covered by the most recent lava flows and have there no evident surface expression (Fig. 1).

| Date | Time | Latitude | Longitude | Depth (km) | Ml |
|---|---|---|---|---|---|
| 17.03.2015 | 123513.800 | 63.81570 | -22.75730 | - | - |
| | 134329.595 | 63.88757 | -22.07062 | 0.135 | 1.68 |
| | 141232.819 | 63.88997 | -22.07200 | 0.135 | 2.07 |
| | 200757.138 | 63.91812 | -21.16431 | 7.499 | 0.41 |
| | 232441.820 | 63.28078 | -24.31998 | 10.005 | 1.97 |
| | 233444.400 | 63.82070 | -22.75670 | - | - |
| 18.03.2015 | 012544.590 | 63.28132 | -24.24640 | 10.005 | 2.41 |
| | 014237.490 | 63.26402 | -24.17299 | 10.005 | 2.04 |
| | 021438.167 | 63.94202 | -21.32619 | 5.553 | 1.11 |
| | 033632.982 | 63.60829 | -23.83504 | 10.005 | 1.5 |
| | 040854.548 | 63.97064 | -20.70711 | 7.071 | 0.81 |
| | 054525.347 | 63.97831 | -20.92498 | 4.52 | 0.61 |
| | 062645.709 | 63.31213 | -24.22322 | 10.005 | 1.62 |
| | 094803.941 | 63.94957 | -21.04885 | 7.004 | 0.45 |
| | 110248.902 | 63.89518 | -21.98165 | 7.688 | 0.4 |
| | 144059.750 | 63.94616 | -21.34695 | 6.344 | 0.85 |
| | 152248.938 | 63.91788 | -22.00966 | 8.244 | 1.03 |

| | | | | | |
|---|---|---|---|---|---|
| | 153855.951 | 63.91488 | -22.01769 | 9.592 | 1.36 |
| | 155817.012 | 63.91555 | -22.01686 | 8.52 | 1.02 |
| 19.03.2015 | 033544.069 | 63.85105 | -22.41394 | 8.5 | 1 |
| | 062524.769 | 63.97066 | -20.54266 | 6.85 | 1.26 |
| | 152428.448 | 63.62228 | -23.48391 | 9.304 | 2.77 |
| | 152549.715 | 63.62851 | -23.48808 | 9.701 | 3.52 |
| | 152809.069 | 63.62165 | -23.49410 | 8.966 | 2.55 |
| | 153958.750 | 63.58426 | -23.35005 | 2.423 | 1.94 |
| | 155620.472 | 63.60349 | -23.29428 | 10.005 | 2.18 |
| | 160151.666 | 63.57256 | -23.38467 | 10.005 | 2 |
| | 221122.717 | 63.93811 | -21.07365 | 5.7 | 0.67 |
| 20.03.2015 | 000533.772 | 63.94842 | -21.76611 | 8.974 | 0.86 |
| | 004942.159 | 63.95584 | -20.23646 | 7.316 | 1.05 |
| | 190350.483 | 63.78902 | -22.81958 | 7.743 | 1.46 |
| 21.03.2015 | 002505.530 | 63.72341 | -23.08535 | 9.274 | 2.4 |
| | 005405.737 | 63.96001 | -20.70190 | 5.956 | 0.35 |
| | 015441.493 | 63.74953 | -23.04996 | 4.461 | 2.33 |
| | 015536.997 | 63.74246 | -23.11171 | 11.35 | 2.14 |
| | 023719.045 | 63.72398 | -22.99030 | 10.617 | 2.08 |
| | 023719.831 | 63.70922 | -22.98475 | 10.059 | 2.14 |
| | 023808.480 | 63.75915 | -23.11457 | 11.252 | 2.05 |
| | 023810.669 | 63.75466 | -23.10264 | 11.774 | 2.56 |
| | 024404.898 | 63.73452 | -23.09302 | 9.282 | 3.24 |
| | 025026.620 | 63.73301 | -23.08797 | 10.417 | 2.28 |
| | 025452.008 | 63.74785 | -23.07770 | 6.987 | 2.22 |
| | 031812.031 | 63.75742 | -23.16852 | 8.602 | 1.65 |
| | 033104.042 | 63.78539 | -22.98797 | 9.873 | 1.67 |
| | 033302.795 | 63.76807 | -22.98377 | 5.032 | 1.87 |
| | 033306.076 | 63.75515 | -23.04505 | 8.533 | 2.25 |
| | 033330.768 | 63.76606 | -23.00832 | 2.616 | 2.21 |
| | 033538.282 | 63.73726 | -23.05952 | 7.768 | 2.87 |
| | 033653.502 | 63.73884 | -23.05099 | 10.165 | 1.99 |
| | 033926.265 | 63.74123 | -23.06257 | 10.617 | 2.13 |
| | 035417.136 | 63.72586 | -23.06321 | 7.942 | 2.46 |
| | 035458.362 | 63.72704 | -23.08016 | 8.689 | 2.56 |
| | 051232.242 | 63.74422 | -23.10125 | 9.838 | 1.29 |
| | 054840.536 | 63.74189 | -23.09292 | 8.934 | 2.15 |
| | 060235.731 | 63.77201 | -22.98975 | 10.64 | 1.72 |
| | 064316.494 | 63.74780 | -23.12904 | 12.887 | 1.62 |
| | 071427.229 | 63.75529 | -23.09480 | 11.416 | 2.1 |
| | 112237.699 | 63.73768 | -23.09242 | 9.926 | 1.54 |
| | 194556.532 | 63.91367 | -21.16575 | 6.05 | 0.28 |
| | 203339.304 | 63.93933 | -21.38728 | 6.635 | 0.68 |
| | 212643.318 | 63.72953 | -23.12332 | 11.88 | 2.29 |

| | | | | | |
|---|---|---|---|---|---|
| 22.03.2015 | 082045.522 | 63.95654 | -21.14264 | 8.743 | 0.29 |
| | 111017.119 | 63.87780 | -22.01269 | 6.608 | 0.36 |
| | 112257.624 | 63.98433 | -20.70193 | 6.388 | 0.39 |
| | 192134.878 | 63.97046 | -21.05165 | 4.913 | 0.26 |
| | 204902.766 | 63.92028 | -21.26286 | 6.782 | 0.35 |
| | 211012.739 | 63.55810 | -23.61405 | 10.005 | 2.19 |
| | 225140.600 | 63.84720 | -22.68880 | - | - |
| 23.03.2015 | 160708.530 | 63.86623 | -22.53574 | 3.563 | 1.4 |
| 24.03.2015 | 003113.952 | 63.97458 | -20.70251 | 5.095 | 0.71 |
| | 061343.553 | 63.66073 | -23.23991 | 13.204 | 1.83 |
| | 062926.968 | 63.63893 | -23.29323 | 9.667 | 2.22 |
| | 063506.798 | 63.65860 | -23.33569 | 11.436 | 1.65 |
| | 063710.401 | 63.67684 | -23.32285 | 8.927 | 2.7 |
| | 064217.452 | 63.73769 | -23.02685 | 13.117 | 2.04 |

**Supplementary Table 1 | List of local earthquakes recorded during the DAS recording period**

| Date | Time (UTC) | Location | Depth (km) | Magnitude Mb | Distance to the fibre optic cable |
|---|---|---|---|---|---|
| 17.03.2015 | 22:12:28 | 135 km NW of Kota Ternate | 44 | 6.2 | 12,300 km |
| 18.03.2015 | 18:27:29 | 75 km NNW from Talcahuano, Chile | 13 | 6.2 | 12,000 km |
| 23.03.2015 | 04:51:38 | 45 km ESE from Putre, Chile | 130 | 6.4 | 10,000 km |

**Supplementary Table 2 | List of Teleseismic earthquakes during the DAS recording period**

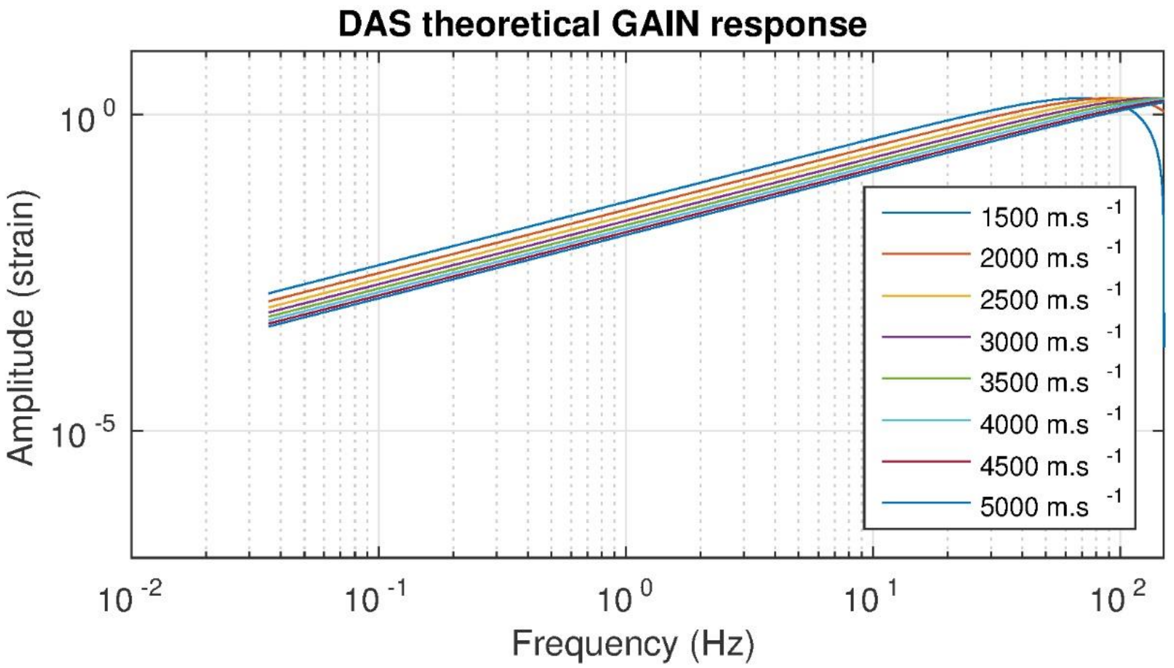


**(a)**

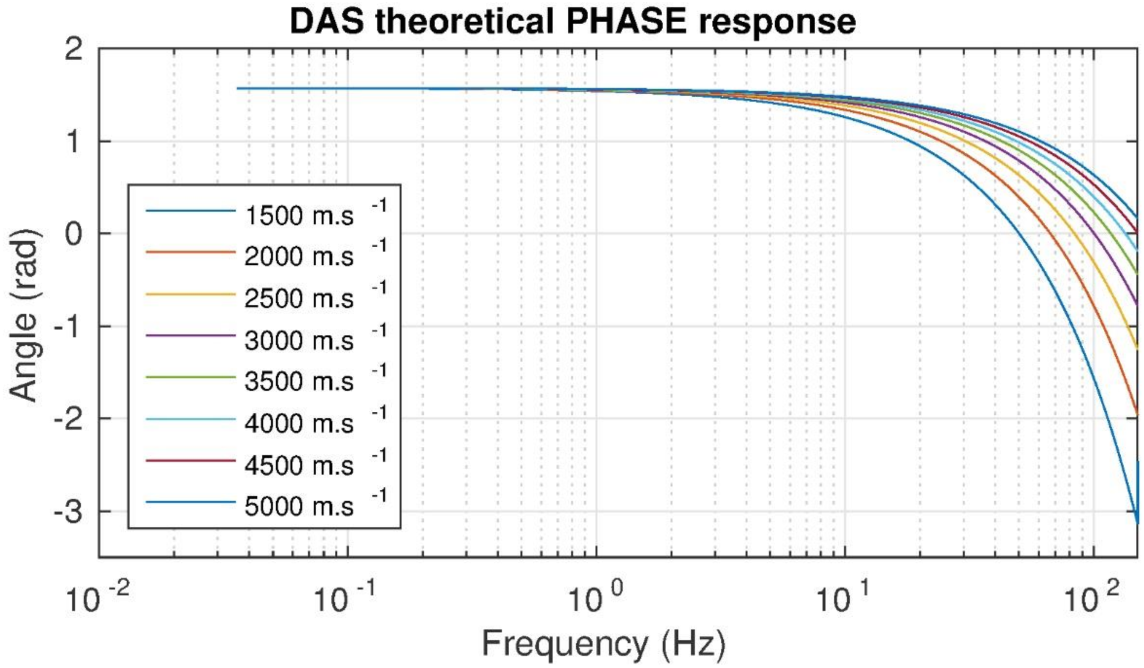


**(b)**

**Supplementary Figure 1 | Theoretical instrumental response (in frequency) to an impulse in the time domain along the cable** (incidence angle = 0; 10 m gauge length). The input signal is a propagating local displacement impulse of 1 nm magnitude along the cable. **a** amplitude; the amplitude output iDAS response is then expressed in strain (nm/m). **b** phase

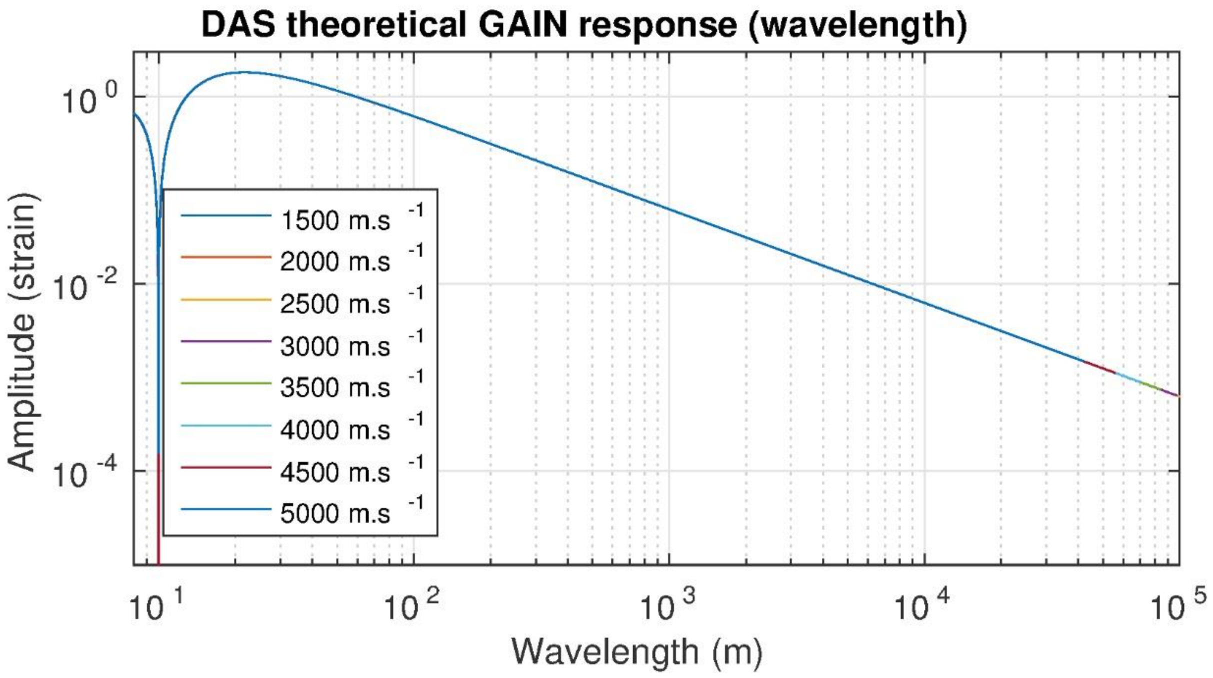


(a)

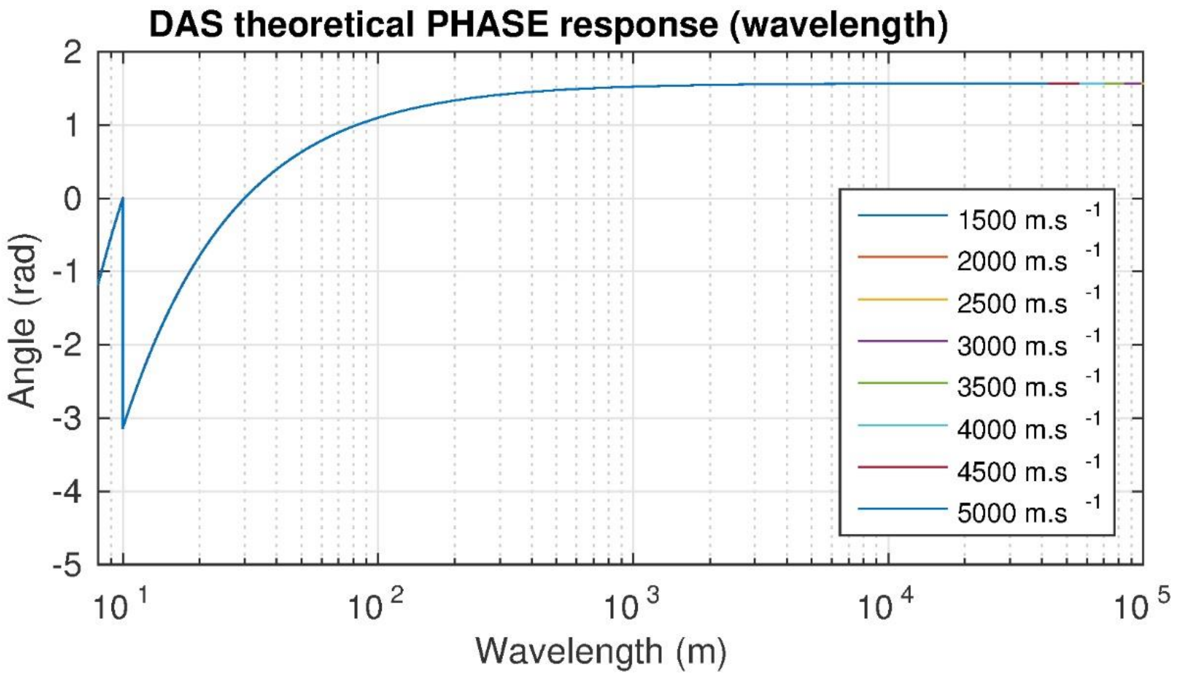


(b)

**Supplementary Figure 2 | Theoretical instrumental response (in wavelength) to an impulse in the time domain along the cable** (incidence angle = 0) expressed in wavelength. There is no dependency with the wave velocity. **a** amplitude. **b** phase

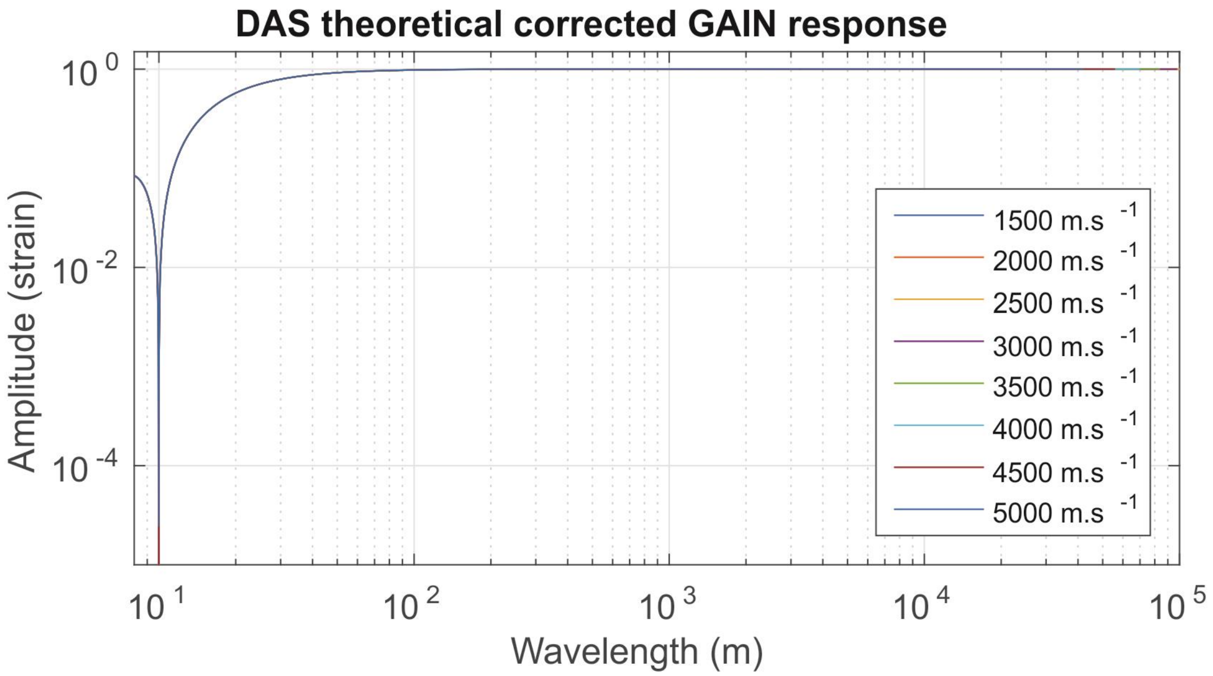


**(a)**

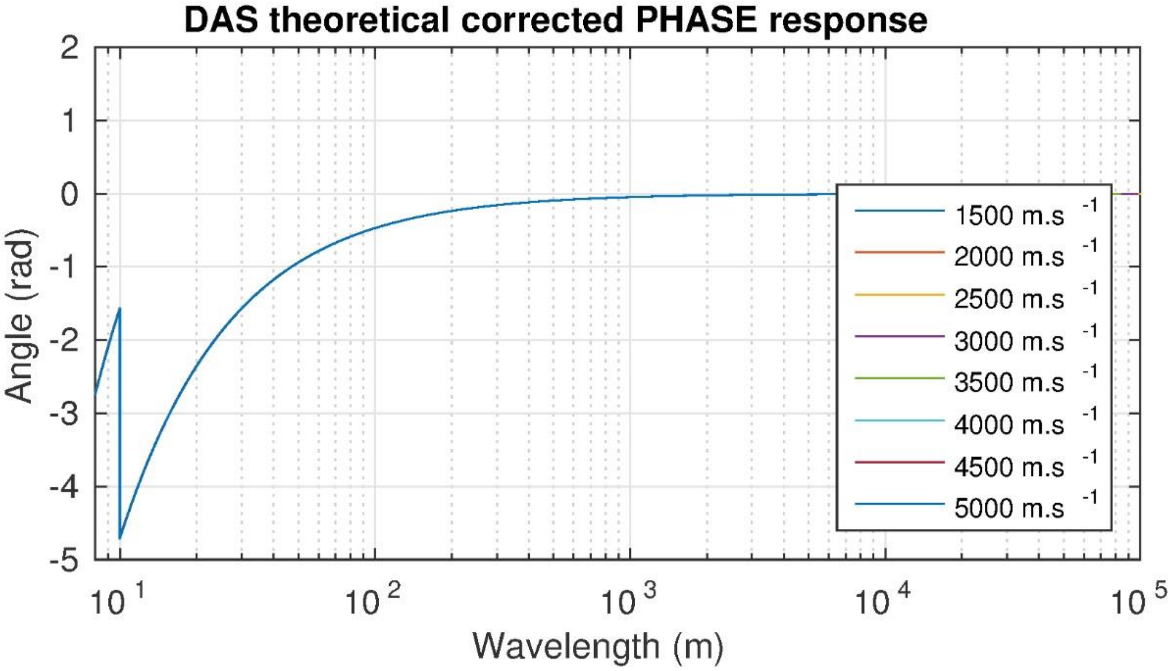


**(b)**

**Supplementary Figure 3 | Theoretical instrumental response corrected from an approximation of the response for the long periods.** The response is flat for wavelengths above 20 m, which is fine for seismic frequencies (< 100 Hz) at velocities above 1500 m/s. In the amplitude plot C=0.0159; in the phase plot the phase shift is π/2. **a** amplitude. **b** phase

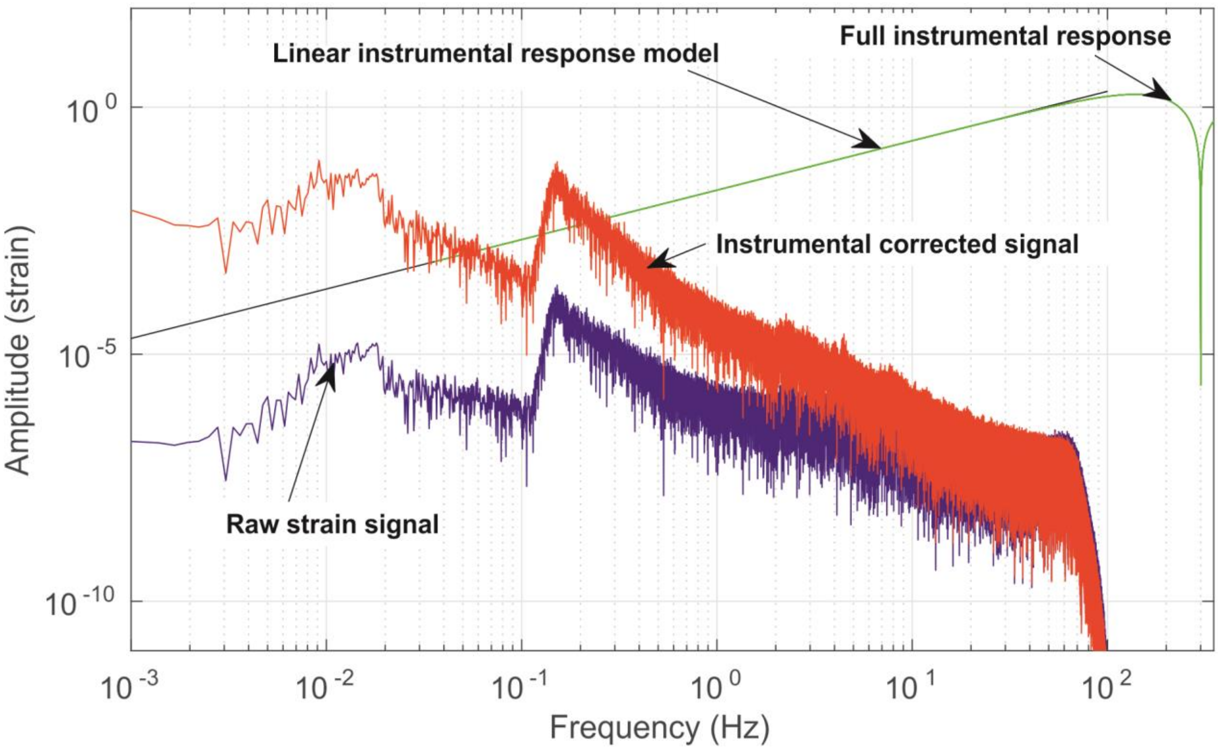


**Supplementary Figure 4 | Restitution of the data by instrumental response correction**

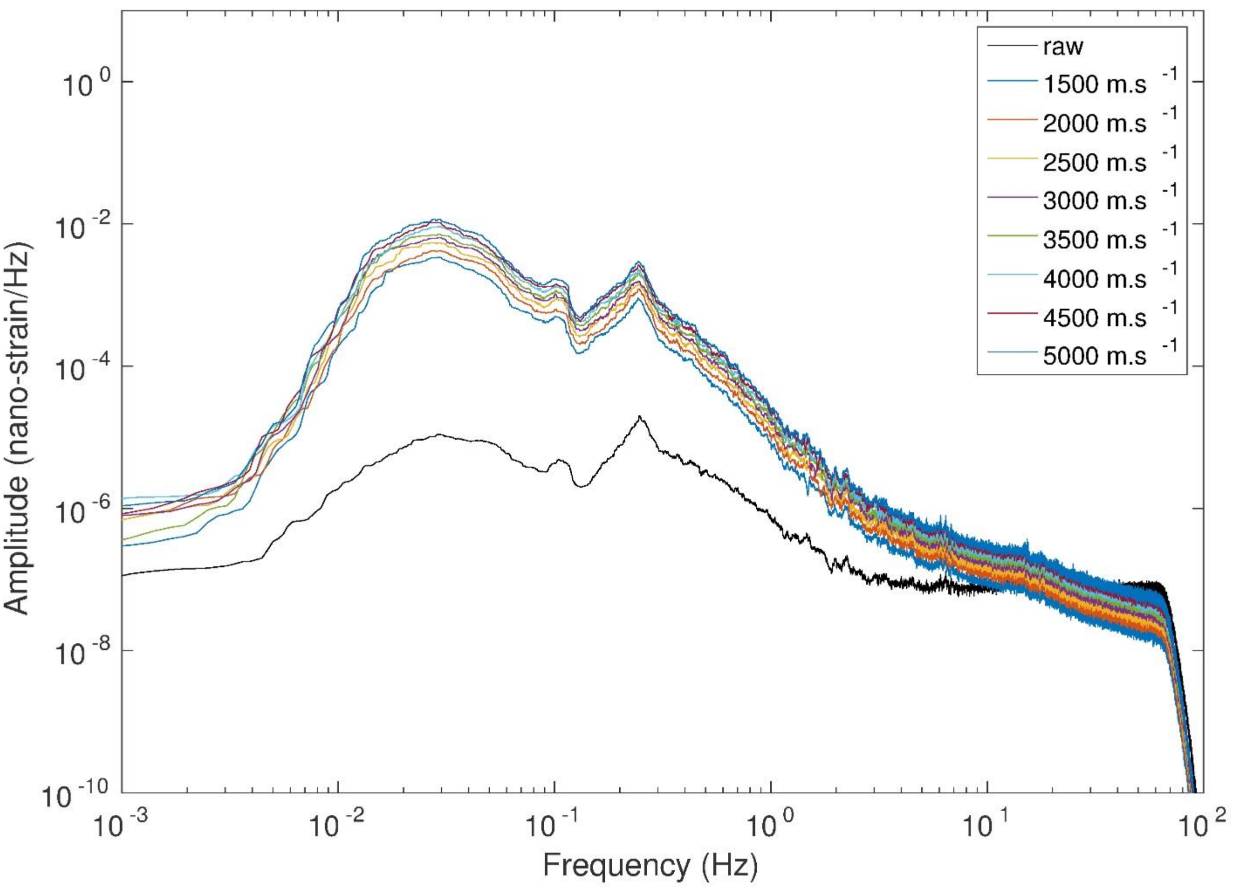


**Supplementary Figure 5 | Restitution for various wave velocities**

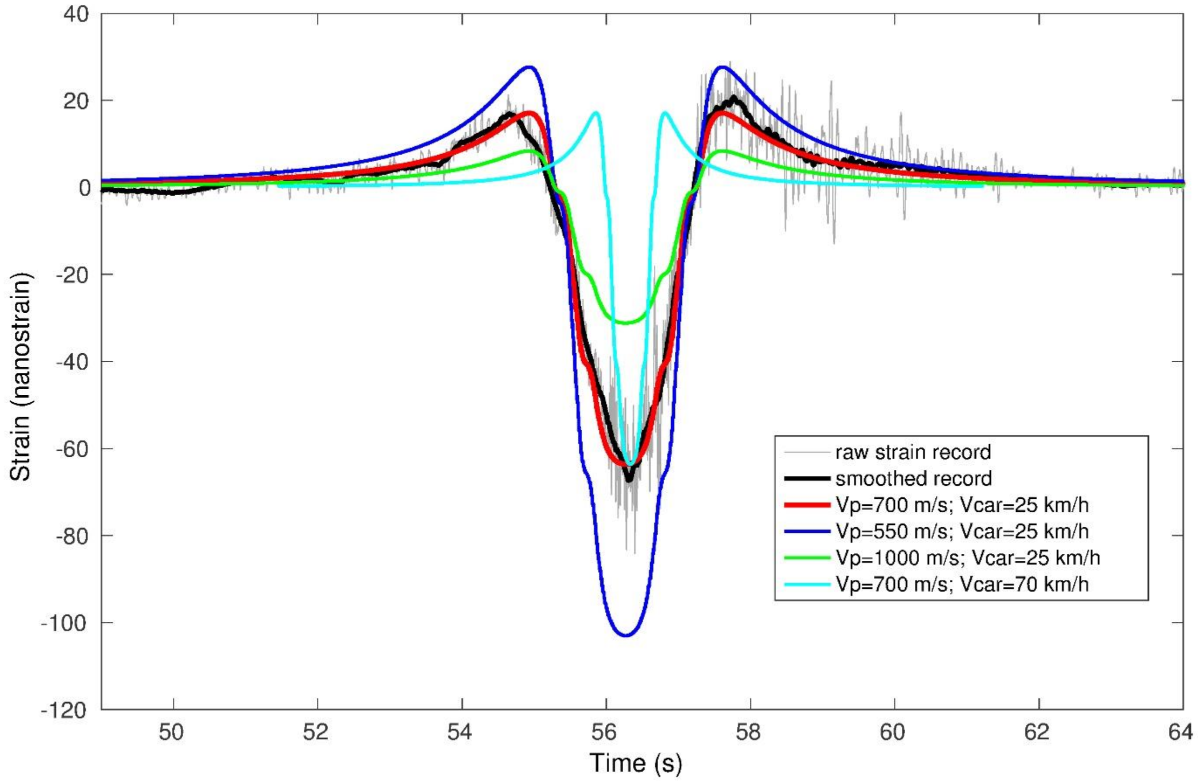


**Supplementary Figure 6 | Deformation of the ground due to a ~2200 kg 4WD car on the ground surface**

We model the weight of the car on the ground as the sum of 4 points load at the surface. The distance between the right and left wheels of the car is taken as~1.6 m and from the front and the rear wheels as ~2.8 m. We assume a ground density of 2000 kg.m$^{-3}$ and a Poisson ratio =0.25, typical values for basalts at the surface[4]. Grey light curve: recorded data. Thick black curve: same data but smoothed. Colour curves: modelled deformation for a car moving at different speed along a road for various P-wave ground velocities. The cable is assumed to be at 0.5 meter depth

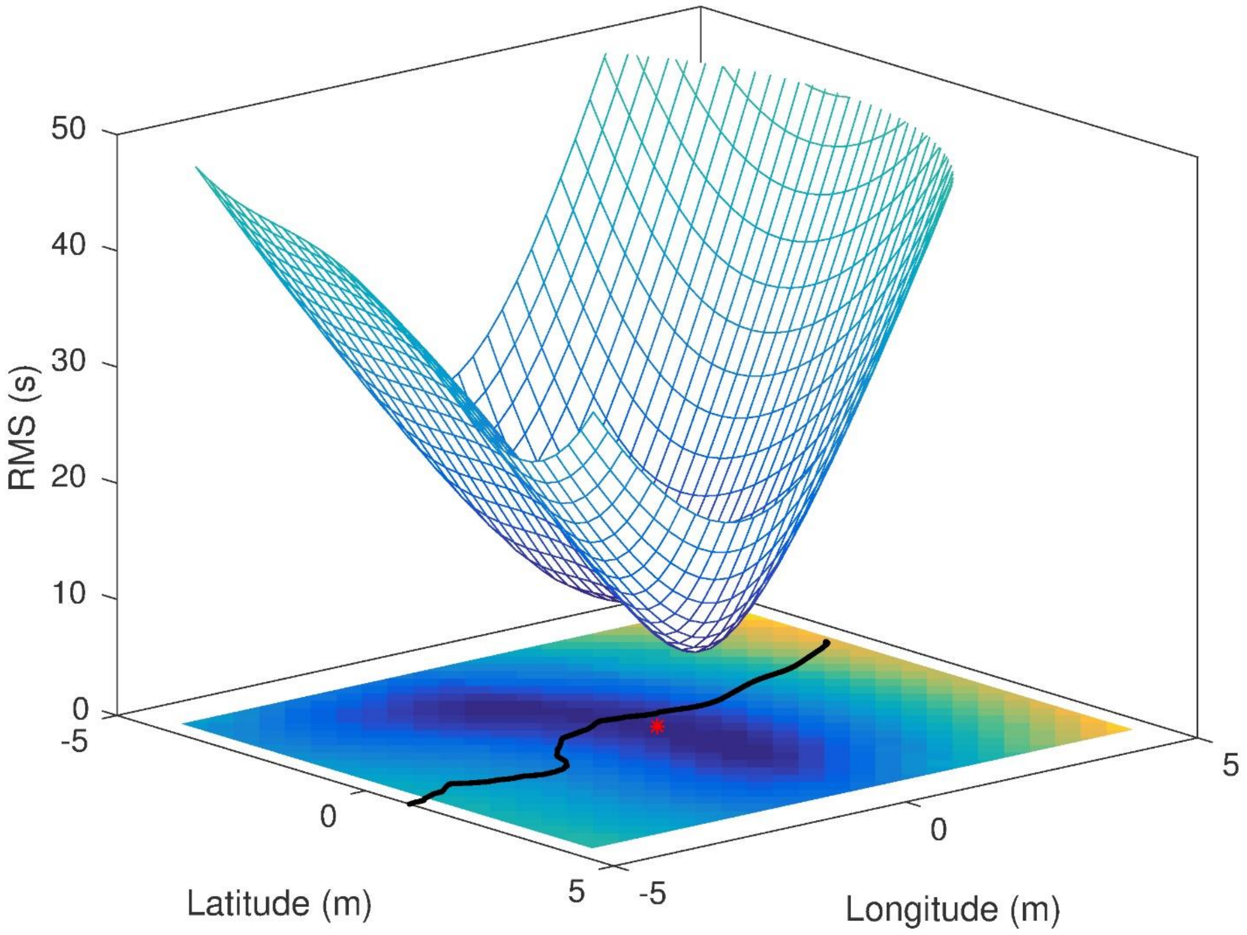


**Supplementary Figure 7 | Probability density function of the hypocentre location determined with DAS data**

We computed the probability density function using a grid search in a 3D grid with 10 km edge. When the hypothetic hypocentre is far from the true hypocentre location, the RMS value increases. When the hypothetic hypocentre is close to the true hypocentre, the RMS value decreases. The minimum value of the misfit is defined as the maximum probability of the hypothetical hypocentre to be at the true hypocentre location. The red star is the position of the hypocentre location defined by the travel time tomography. When projected on the base plane, the lowest values of the pdf (deep blue) encompass the most probable hypocentre location, similar to the hypocentre location found with the 3D tomographic inversion. The black line is the location of the fibre-optic cable. The inversion was performed with about 3000 P- and S- picks along the cable

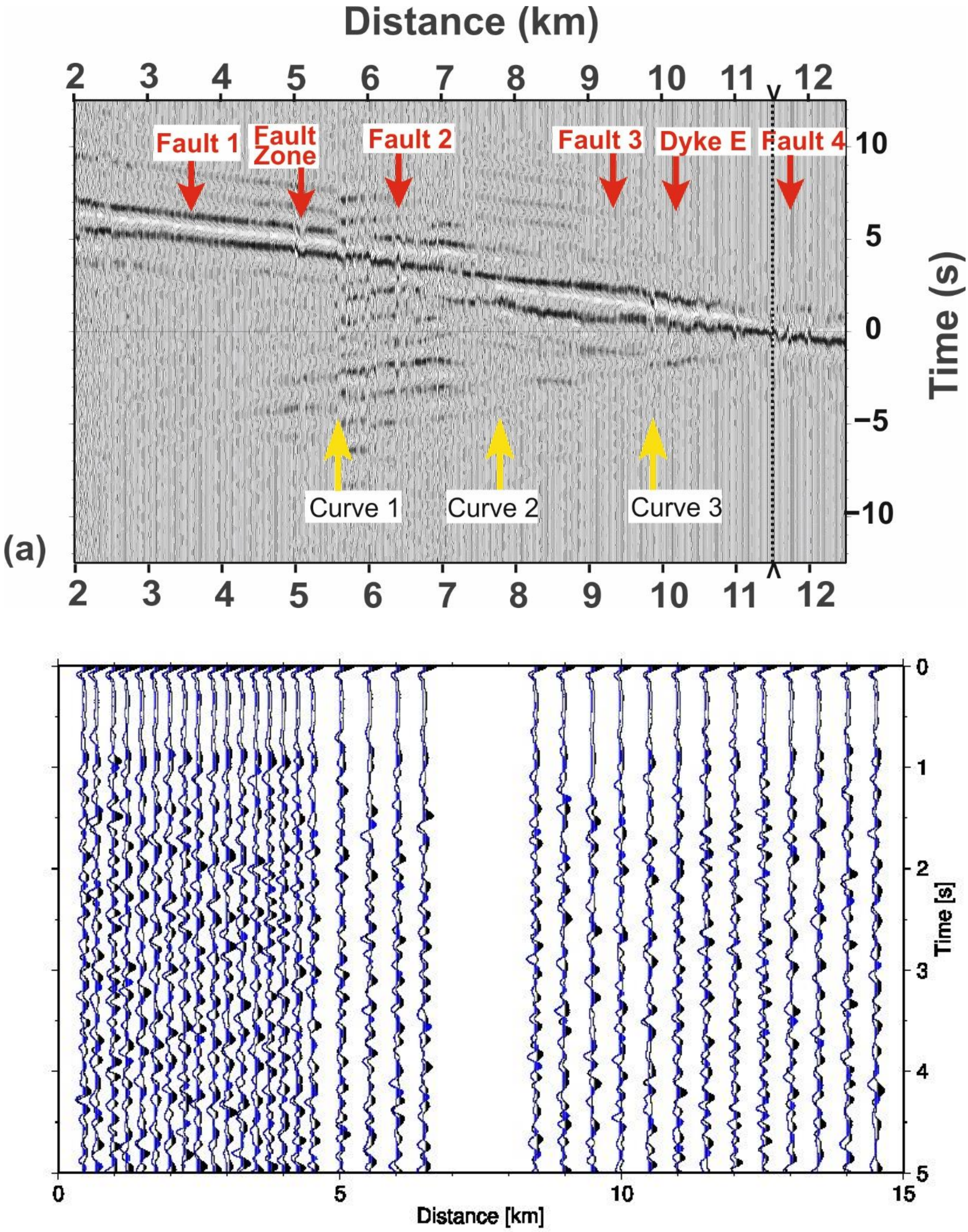

Distance (km)
2 3 4 5 6 7 8 9 10 11 12
Fault 1
Fault Zone
Fault 2
Fault 3
Dyke E
Fault 4
10 5 0 −5 −10
Time (s)
Curve 1
Curve 2
Curve 3
(a)
0 1 2 3 4 5
Time [s]
0 5 10 15
Distance [km]


(a)

(b)

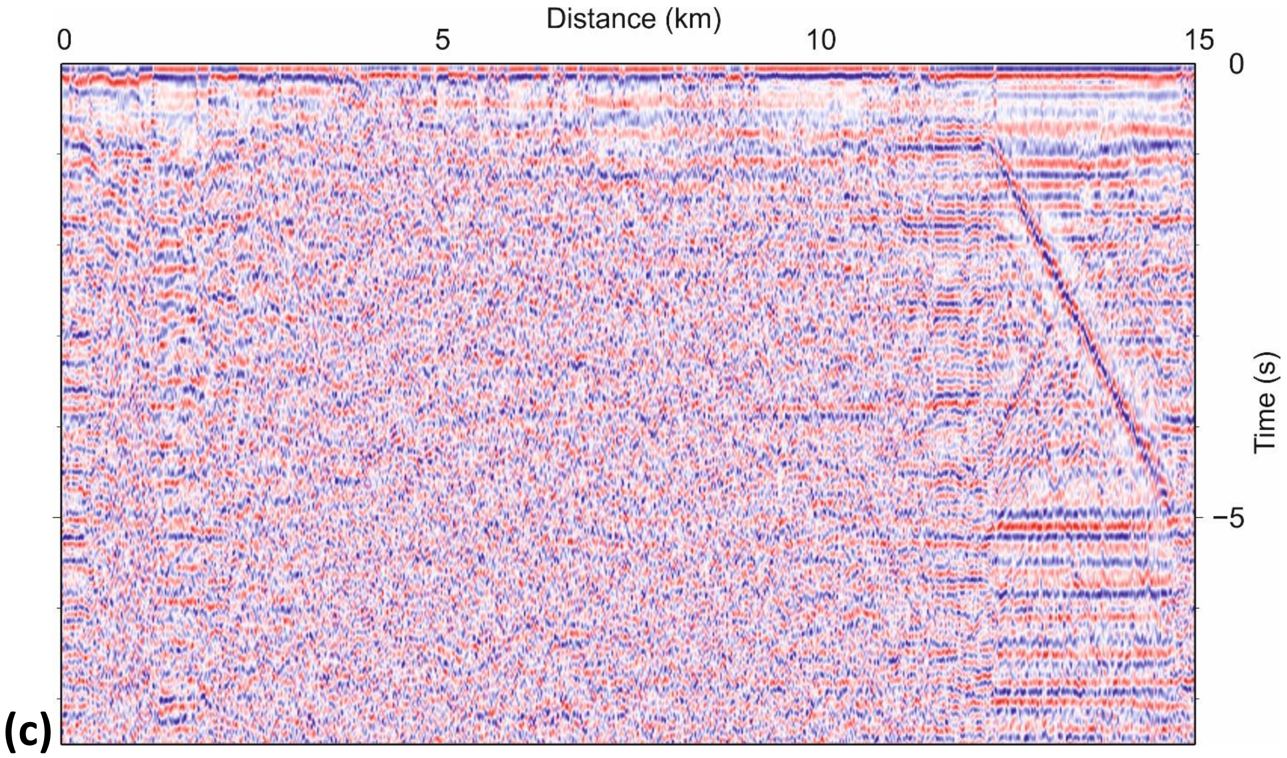


**Supplementary Figure 8 | Ambient noise techniques results with DAS records.**

**a** Ambient noise based, cross-correlation computed between all traces of the cable with respect to one arbitrary trace (at position ~11.5 km). The traces have been band-pass filtered between 0.5 and 2 Hz to enhance the Rayleigh waves. Note the perturbation of the wave field due to the presence of several geological features including a fault damage zone (FDZ) at ~5 km. **b** Autocorrelations computed for selected DAS/DVS traces (black) and for their closest geophones (blue) deployed along the cable (Fig. 1) showing that both sensors types led to comparative correlation results. Horizontal components of the geophones have been rotated into the direction along the optical cable. An automatic gain control (scaling in a moving window) with a window length of 1 second has been applied to suppress the strong arrival at 0 seconds. For clarity, the geophone traces have been shifted by few meters to the right. **c** Auto-correlation for all traces of the fibre optic cable, indicative for a highly complex, fine-scale structure below the fibre. Note a slight perturbation of the amplitudes in the image at the distance ~5 km, corresponding to the position of the fault damage zone and at ~10 km corresponding to the location of a dike. Beyond distance ~12 km, strong man-made noise (pipeline) dominates the autocorrelation signal

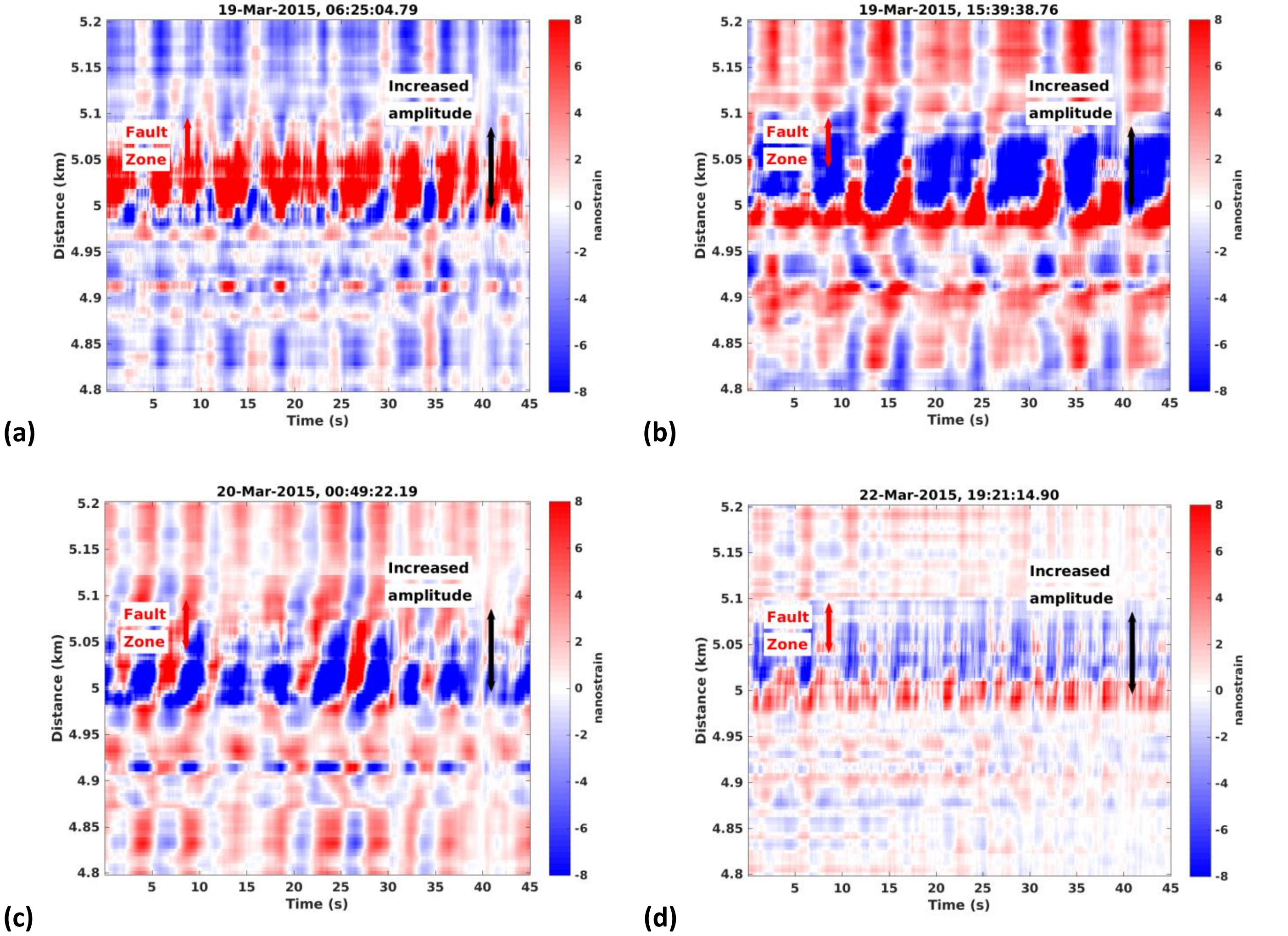


**Supplementary Figure 9 | Examples of trapped micro-seism waves at various recording times (strain).**

The fault zone visible at the surface in the field is indicated with the red double-arrow; the area with increased micro-seism amplitude is indicated by the black double-arrow. **a** 19 March 2015 06:25:4.79. **b** 18.03.2015 15:39:38.76. **c** 20.03.2015 00:49:22.19. **d** 22.03.2015 19:21:14.90

**Supplementary References**

1. Sigmundsson, F., Einarsson, P., Hjartardóttir, A.R., Drouin, V., Li, S., Jónsdottir, K., Árnadottir, T., Geirsson, H., Hreinsdóttir, S. & Ófeigsson, G. Geodynamics of Iceland and the Signatures of Plate Spreading. *Special Issue J. Volcanol. Geotherm. Res. – "Reykjanes"*, **in press**.

2. Sigmarsson, O. & Steinthórsson, S. Origin of Icelandic basalts: a review of their petrology and geochemistry. *J. Geodynamics* **43**, 87-100 (2007).

3. Flovenz, Ó.G., & Saemundsson, K. Heat flow and geothermal processes in Iceland. *Tectonophysics* **225**, 123-138 (1993).

4. Clifton, A. E. & Schlische, R. W. Fracture populations on the Reykjanes Peninsula, Iceland: Comparison with experimental clay models of oblique rifting. *Journal of Geophysical Research* **108**, B2, 2074, doi:10.1029/2001JB000635 (2003).

5. Jumikis, A. R. Rock mechanics. *Trans. tech Publications*, Clausthal (1979).
