# Supplementary material for: Dynamic strain determination using fibre-optic cables allows imaging of seismological and structural features
Source: Nat Commun. 2018 Jul 3;9:2509. doi: 10.1038/s41467-018-04860-y (PMC6030137; doi:10.1038/s41467-018-04860-y)
Supplement: Supplementary file 2 — Peer Review Report [file 41467_2018_4860_MOESM2_ESM.pdf]

Reviewers' comments:

Reviewer #1 (Remarks to the Author):

The article describes the use of distributed acoustic sensing of telecommunication fiber to perform seismic monitoring in Iceland. I should note I am not a seismologist, but I have worked quite a bit with DAS strain sensing.
The article presents some really interesting results and proposes some future applications of DAS. Prior work has shown that DAS can be used with telecommunication fibers to sense ground motion, but this article breaks new ground on a few fronts. First, the geology they monitor is more interesting with clear faults that are traversed by the fiber optic cables. Seismic waveforms trapped between faults are clearly identified. Second, the strain aspect of the data are elaborated in greater detail than this previous reporting by Dou et al.
Unfortunately, I the presentation is currently such that the article is not ready for publication in Nat. Comm. The authors try to put too much into the article forcing them to rely upon prior knowledge and jargon that makes the article truly accessible only to seismologists. I think there were aspects that could have been removed such as the acquisition of velocity using the car movement. Seismic sections are note accessible to the larger scientific community and the many arrows only confuse matters. What are the symbols in Figure 4b? The figures need to be simplified and cleaned up at minimum. I suggest the author's think about ways to convey these results to a larger audience.
Also, although the strain aspect is interesting, I do not think it is sufficiently developed to include wholly in the article. It was not clear to me how they obtained "permanent" strain from the data, for example. Strain is obtained from strain rate through local integration (i.e. multiply by time step), then displacement is obtained by integrating with space (i.e. multiplying by gauge length). Thus, displacement changes through time (see Becker et al., cited in the article, for example). How is the strain known to be permanent? I'm not arguing with the interpretation, I just can't follow the process they used to arrive at figure 5 or the interpretation. While they are able to confirm the acceleration measurements, the strain measurements come purely from data processing of the apparent strain rate on the fiber. I don't think there is sufficient evidence to make strong conclusions about strain measurements.
I don't understand the apparent play on words with "bright" future. Is this a seismic term or because we are working with light? Similarly, in the conclusions, I think the proposal of "strainology" is a little cute and unjustified.
My suggestion is that the authors focus the results on the imaging of faults obtained by the DAS telecommunication network. As they point out, you would not see wave trapping without the density provided by the fiber monitoring. It would be interesting, I think if they could compare geophone and DAS results side by side to show how the geophones miss the faults. This would make the point clearly. It would also be interesting if they could demonstrate that they located more faults along the fiber path. Currently, figure 5 mixes displacement and wave trapping which is overwhelming for the reader, or at least this reader.
The material summarized in this article is worth of publication in Nat Comm and might be of interest to readers if it were presented in a more accessible manner.

Reviewer #2 (Remarks to the Author):

Please see the uploaded file "Rev-DAS_DVS-Nature.pdf"

Editorial Note: Rev-DAS_DVS-Nature.pdf Part 1 included below

To Authors

First, I would like to offer my congratulations to the authors for performing some important research, and for writing a potentially impactful paper.
The paper presents some interesting, though preliminary, results of analyzing seismic data obtained by using fibre-optic recording of seismic waves (DAS/DVS). This technology is most likely to have a profound impact on seismology because it enables to densely sample wavefields with large arrays at a reasonable cost.
In general, the paper is well and clearly written. Some detailed comments and suggestions are contained in the annotated PDF file that I upload with the review.

However, I think that two issues must be addressed before the paper is published.

1) All through the paper (introduction, results, conclusions) a clear distinction must be made on what could be (in principle) achieved with fibre-optic recording technology and what is actually demonstrated from the data the authors have collected and are presenting in the paper. Whereas it is reasonable to assume that large arrays employing fiber-optic cable will eventually achieve all the goals listed in the paper, these goals are not actually achievable from the data and the analysis presented in the paper. The presence of faults is detected (or may be inferred from anomalies in the seismic recording) not imaged and characterized. Hypocentres location cannot be uniquely determined by using a single-component sensor array with quasi-linear geometry. This kind of arrays cannot resolve ambiguities with respect to the angle of incidence on the plane perpendicular to the arrays itself. In my opinion, the final version of the paper must make these distinctions carefully and clearly.
2) The data-collection experiment presented by the authors is one of the first, but not the only experiment that recorded seismic data using fiber-optic cables leveraging telecommunication infrastructure. Below you can find references to 5 other publications, listed in chronological order, that I cut and pasted from the web. These publications report about two other data-collections experiments that use fibre-optic cables placed in telecommunication conduits. Please notice that the first one was actually published a few weeks before the first reference describing the experiment reported in this paper. I think that a subset of these publications should be referenced in the paper.

From EAGE website

Continuous Subsurface Monitoring by Passive Seismic with Distributed Acoustic Sensors - The "Stanford Array" Experiment
Authors: E.R. Martin, B.L. Biondi, M. Karrenbach and S. Cole
Event name: First EAGE Workshop on Practical Reservoir Monitoring
Session: Improving Cost Effectiveness Through Alternative Technologies
Publication date: 06 March 2017
DOI: 10.3997/2214-4609.201700017
Info: Extended abstract, PDF (2.53Mb)
Language: English

Continuous Subsurface Monitoring by Passive Seismic Data Recorded with Distributed Acoustic Sensors - The "Stanford DAS Array" Experiment
Authors: E. Martin, B.L. Biondi, S. Cole and M. Karrenbach
Event name: 79th EAGE Conference and Exhibition 2017
Session: Time Lapse and PRM I

Publication date: 12 June 2017
DOI: 10.3997/2214-4609.201700812
Info: Extended abstract, PDF (2.62Mb)
Language: English

Ambient Noise Interferometry from DAS Array in Underground Telecommunications Conduits

Authors: E. Martin, B. Biondi, M. Karrenbach and S. Cole
Event name: 79th EAGE Conference and Exhibition 2017
Session: Passive Seismic - Ambient Noise, Methods and Case Studies
Publication date: 12 June 2017
DOI: 10.3997/2214-4609.201700743
Info: Extended abstract, PDF (1.73Mb)
Language: English

From AGU website (presented at the December AGU meeting in New Orleans)

Dark Fiber and Distributed Acoustic Sensing: Applications to Monitoring Seismicity and Near-Surface Properties

Jonathan Blair Ajo Franklin[1], *Nate Lindsey*[2], *Shan Dou*[2], *Barry M Freifeld*[2], *Thomas M Daley*[3], *Chris Tracy*[2] *and Inder Monga*[2], *(1)Lawrence Berkeley National Laboratory, Geophysics, Berkeley, CA, United States, (2)Lawrence Berkeley National Laboratory, Berkeley, CA, United States, (3)Lawrence Berkeley Lab, Berkeley, CA, United States*

Earthquake recording at the Stanford DAS Array with fibers in existing telecomm conduits

Eileen Rose Martin[1], ***Biondo C. Biondi***[1], *Siyuan Yuan*[2], *Steve Cole*[3] *and Martin Horst Karrenbach*[3], *(1)Stanford University, Stanford, CA, United States, (2)Stanford University, Civil Engineering, Stanford, CA, United States, (3)OptaSense, Inc, Brea, CA, United States*

Editorial Note: Rev-DAS_DVS-Nature.pdf Part 2 included below
Editorial Note: In their review of the first version of this manuscript, reviewer 2 added their comments to the manuscript file. These comments, excluding minor textual revisions, have been copied into this Peer Review File.

On line 99: “buried how? Trenched? in conduit? This is important because of effects on ground-cable coupling. Please be specific.”

On line 163: “This claim is puzzling, and probably misleading. From the quasi-linear fibre cable you cannot resolve the incidence angle in the plane orthogonal to the cable. Therefore, an independent hypocentre localization using only the fibre data would not be able to resolve that ambiguity.”

On line 170: “I have some doubts about the accuracy of this claim too. The events shown in Figure 4d are most likely related to propagation taking place very close to the surface or even in the air (apparent velocity is measured about 200-300 ms (line 365 in text) . Therefore the analysis of these events will not provide much information on the fault zone in the subsurface.”

On line 194: “Figure 2c should be zoomed in around few arrivals for being informative. As it is, it just shows that there is a correspondence between the recording time at the broadband station and at the fibre cable. Phases cannot be compared at this scale, and even amplitudes are difficult to compare.”

On Figure 3, Page 10: “In the figure is impossible to distinguish the two green curves. One of the two should be plotted with a different colour (magenta may work here)”

On line 287: “This is a huge range for being really helpful to confirm accuracy of the estimates from the DAS/DVS data.”

On line 299: “Same problem as discussed in my comment at line 163 in the text. Given the quasi-linear geometry of the fibre cable, the hypocentre cannot be located without ambiguities. It is true that using a fire array with fully 2D geometry (e.g. cables crisscrossing along two orthogonal directions) we could locate hypocentres, but it is not the case here.”

On line 322: “Too little information in the data to claim that you can characterise structural features. Detection yes, characterization, no.”

On line 341: “This is mostly one-sided correlation because ambient noise has a strong directivity. Say so, and relate the reasons of the noise directivity with know geophysical reasons.”

On line 349: “Imaging is too strong of a word here. What about "analysis"”

On line 358: “See comment at line 169 in the text. My guess is that these "trapped phases" do not propagate at depth, but close to the surface, or even in the air.”

On line 365: “This is fairly close to sound velocity in air (331 m/s at 0 degree Celsius)”

On line 406: “Not really unreported yet. See my comments in the "comments to Author" on references that should be added.”

On line 414: “See previous comments on hypocentre localisation.”

On line 415: “See previous comments. You cannot exclude that these events are related to propagation at the surface, not in the subsurface.”

On line 430: “Do you mean "hazard exploration"? It reads like that. Would "identification" a better word?”

On Figure S1, page 38: “These graphs probably assume a fixed gauge length. Say which gauge length you are assuming.”

On line 930: “You are probably assuming that waves move in the direction of the cable. Please say so explicitly.”

On line 940: “The phase response is not linear above 20 m. You may approximate as linear, that is OK, but do not say that that is linear!”

On line 986: “What does that mean? Did you mean with lower elastic properties of the rocks?”

On Figure S8a, page 43: “I can see events in which the blue and black traces are in good agreement, but also events for which are completely out of phase. In my opinion, no strong conclusions can be drawn from this figure!”

On line 1002: “What does cross-crossing mean?”

Answer to reviewer 1 comments:

The article describes the use of distributed acoustic sensing of telecommunication fiber to perform seismic monitoring in Iceland. I should note I am not a seismologist, but I have worked quite a bit with DAS strain sensing.

The article presents some really interesting results and proposes some future applications of DAS. Prior work has shown that DAS can be used with telecommunication fibers to sense ground motion, but this article breaks new ground on a few fronts. First, the geology they monitor is more interesting with clear faults that are traversed by the fiber optic cables. Seismic waveforms trapped between faults are clearly identified. Second, the strain aspect of the data are elaborated in greater detail than this previous reporting by Dou et al.
Thank you for this appreciation. This comforts us to resubmit a revised and improved version of our manuscript.

Unfortunately, the presentation is currently such that the article is not ready for publication in Nat. Comm. The authors try to put too much into the article forcing them to rely upon prior knowledge and jargon that makes the article truly accessible only to seismologists.
In general, we believe we have produced a new version where we have better explained our observations and included some basic principles of seismology in order to make the non-seismological reader more receptive to the power of the method.

I think there were aspects that could have been removed such as the acquisition of velocity using the car movement.
We think that the car approach is an elegant way to validate the ability of DAS to measure strain properly in a quantitative way at low frequencies, and that the deformation measured fits very well with a simple model. This example illustrates the unprecedented fact that with both a car and a telephone line (i.e. fibre optic) it is now possible to probe sub-surface parameters interesting for exploration of the Earth and useful for seismic hazard assessment, which is certainly of great interest for non-seismologists. As the reviewer 2 did not complain, we wish to keep this figure. However, in order to comply as much as possible with the suggested remarks and simplify the main text to make the message clearer, we propose to put the car observation and modelling as supplementary material and we have changed the color of the curves to make the figure more readable.

Seismic sections are note accessible to the larger scientific community and the many arrows only confuse matters. What are the symbols in Figure 4b? The figures need to be simplified and cleaned up at minimum. I suggest the author's think about ways to convey these results to a larger audience.
We did indeed simplified the figure 4 (new figure 3) with a more focus aspect. In order to further simplify the figure, we moved the seismic cross-correlation results in the appendix (to the ambient noise topic which is now fully in supplementary information). We also added a legend for the symbols in figure 4b (now figure 3c), in addition to the description of the symbols in the figure caption. By doing so, figure 3 is more focused on structural features using only the earthquake; this indeed simplify the main text of the paper. Thanks to the reviewer for such comment. Therefore the old figure 4 a, b, d (now figure 3) makes the focus in a story in its own: crustal exploration with 1 earthquake record.

Also, although the strain aspect is interesting, I do not think it is sufficiently developed to include wholly in the article. It was not clear to me how they obtained "permanent" strain from the data, for example.
The term "permanent" is certainly excessive. We removed this term, and replaced it by indicating that strain for some traces show an offset prior to its initial value and does not relax for at least several minutes, which is indeed not necessarily "permanent", but rather quasi-static.

Strain is obtained from strain rate through local integration (i.e. multiply by time step), then displacement is obtained by integrating with space (i.e. multiplying by gauge length). Thus, displacement changes through time (see Becker et al., cited in the article, for example).

Indeed Becker et al uses the gauge length as an integrator value for the displacement computation. A rigorous definition for the integration of strain into displacement would be to use the spatial sampling rate, which is 4 m in our case, and not the gauge length. However, each strain observation is obtained as an average value integrating observations over a distance wider than the gauge length. The exact way on how to obtain the displacement accurately is beyond the scope of the paper and could be a subject of an entire specific study. Therefore, we recomputed the figure using the suggestion of the reviewer (the gauge length as in Becker et al, and indicate that the choice of the integration length is matter of research.

How is the strain known to be permanent?
We cannot say indeed this is permanent, but exhibit clearly a significant shift from its initial position. We rephrased accordingly.

I'm not arguing with the interpretation, I just can't follow the process they used to arrive at figure 5 or the interpretation.
We detailed our computation procedure more accurately (in methods: Strain and displacement and velocity determination) showing that there is no artefact in the computation.

While they are able to confirm the acceleration measurements, the strain measurements come purely from data processing of the apparent strain rate on the fiber.
We did not measure or compute any acceleration; the seismometers we use measure velocity. We integrated the velocity to obtain relative ground displacement, which is therefore coming from data processing. While performing the DAS measurements, we measure strain rate, which when integrated in time gives strain, which can also be seen as a relative displacement, when integrated in space, we obtain displacement. In figure 2e, we indeed validate both procedures. All results come from data processing.

I don't think there is sufficient evidence to make strong conclusions about strain measurements.
We explain better how we computed the strain; note that strain computation is performed by many other authors in the same way as ours and yet the results are not discussed as we do. Our results are coming out from the data and simple computation such as integration. When a seismometer records velocity, we are able to integrate in time to obtain displacement. We do the same here: integration of strain rate produces strain. The process is the same for all traces, so if they behave differently it can be for specific reasons for those anomalous traces that exhibit strain steps. However, we note that most of those traces with strain steps are located exactly at known faults. The bouncing waves (reflections) allow to find hidden faults, and therefore we suggest that other traces showing similar strain shifts, could correspond to faults.

I don't understand the apparent play on words with "bright" future. Is this a seismic term or because we are working with light?
Our intention was certainly not to play with the word, and we a sorry the reviewer had such thought. Possibly, this was generated by the quotes. Therefore we propose to remove them.
According to the Oxford dictionary, there are several meaning to the word "bright", below are the one that applies to the DAS technology:

1. "giving out or reflecting much light". This is adapted to the DAS technology, as it is using the light and what is measured is a back-scattered (e.g., reflecting) light.
2. "intelligent and quick-witted". As an example "bright idea" is given. We believe that the so intense excitement the scientific community is bringing to the DAS technology, the idea of using is rather bright…

3. There is also an example of "bright light", which is therefore a combination of the senses, which makes it really adapted to the DAS technology.
4. Another expression talks about "bright future". We use the light in our measurement and by doing so we will obtain more information about the fault structure and dynamics making the future of seismology brighter than before. As there may be other futures for seismology, we opted for "a" future.

However, we may also propose another title ""Fibre optic sensing brightens seismological Earth exploration and hazard assessment". The final title could be the following after discussion with the Associate Editor: **A vibrant future for seismology by using fibre-optic cables**

Similarly, in the conclusions, I think the proposal of "strainology" is a little cute and unjustified.
We agree with the reviewer, removed this word and rephrased.

My suggestion is that the authors focus the results on the imaging of faults obtained by the DAS telecommunication network. As they point out, you would not see wave trapping without the density provided by the fiber monitoring. It would be interesting, I think if they could compare geophone and DAS results side by side to show how the geophones miss the faults. This would make the point clearly.
We added a figure with the earthquake recorded by the geophones (Figure 3a). We clearly see in the geometry of the geophone array that the fault is missed, and that no geophone was not located close enough to the fault zone.

It would also be interesting if they could demonstrate that they located more faults along the fiber path.
We could find evidence in many figures that the records are disturbed locally, where there are visible faults at the surface. These locations are indicated, e.g., by arrows in figure 3d. We added details of several of the records in the supplementary information, focusing on only one example in the main text to keep the demonstration simpler and the paper more readable. However, we left the arrows and changed figure caption and made their meaning clearer.

Currently, figure 5 mixes displacement and wave trapping which is overwhelming for the reader, or at least this reader.
We agree that the initial figure was somewhat too rich. We separated both results: figure 4 focuses now on structural features that are obtained from the analysis of records of 1 earthquake and the trapped waves and figure 5 addresses sudden localized strain jumps and displacement for inferring creeping processes.

The material summarized in this article is worth of publication in Nat Comm and might be of interest to readers if it were presented in a more accessible manner.
Thanks a lot for this comment, which we appreciate very much! We have worked out the comments to make it more accessible to many readers.

Answer to reviewer 2 comments:

First, I would like to offer my congratulations to the authors for performing some important research, and for writing a potentially impactful paper.
Thanks a lot for this encouraging comment and prognostic for our research.

The paper presents some interesting, though preliminary, results of analyzing seismic data obtained by using fibre-optic recording of seismic waves (DAS/DVS). This technology is most likely to have a profound impact on seismology because it enables to densely sample wavefields with large arrays at a reasonable cost.
We agree completely with this assessment. Our manuscript aims at demonstrating this.

In general, the paper is well and clearly written. Some detailed comments and suggestions are contained in the annotated PDF file that I upload with the review.
We added comments in the pdf file that was commented by reviewer 2. And modified accordingly in the docx text.

Editorial Note: In their response to reviewer 2, the authors added their comments to the manuscript file. These comments, excluding minor textual revisions, have been copied into this Peer Review File, and can be found on pages 12-13.

However, I think that two issues must be addressed before the paper is published.
1) All through the paper (introduction, results, conclusions) a clear distinction must be made on what could be (in principle) achieved with fibre-optic recording technology and what is actually demonstrated from the data the authors have collected and are presenting in the paper. Whereas it is reasonable to assume that large arrays employing fiber-optic cable will eventually achieve all the goals listed in the paper, these goals are not actually achievable from the data and the analysis presented in the paper. Fair enough. We have in our corrections kept that in mind. For example, we introduced "may" or "might" where we extrapolate our results and we mentioned more clearly "we demonstrate" or "we show" when it concerns our data and results.

The presence of faults is detected (or may be inferred from anomalies in the seismic recording) not imaged and characterized.
We agree there is a difference. We indeed obtained an estimation of the velocity in the fault zone, which is the way we detected the faults, but we have not a detailed structure or characterized in detail.

Hypocentres location cannot be uniquely determined by using a single component sensor array with quasi-linear geometry. This kind of arrays cannot resolve ambiguities with respect to the angle of incidence on the plane perpendicular to the arrays itself. In my opinion, the final version of the paper must make these distinctions carefully and clearly.
The hypocenter presented in the manuscript is obtained from a grid search from travel times that were computed from ray tracing. The single component is indeed a limitation, but not in the present case: P- and S-waves would be visible even on single component geophone and also on the DAS records, although single component, and are therefore sufficient to pick phase's arrival times. As the cable is not a straight line (see figure 1), but has curves, the array (although not optimal) is still able to infer a location, which makes sense. Thus, we obtain a probability density function (as indicated in the first manuscript). To make it very clear we plotted the pdf in an additional Supplementary figure. In the figure, we clearly see that the pdf is indeed elongated in the direction perpendicular to the cable, which is indeed not surprising, there was no doubt in our mind about this. However, the pdf results reveal a minimum at the hypocenter location. We included such clearer statements in the revised manuscript.

2) The data-collection experiment presented by the authors is one of the first, but not the only experiment that recorded seismic data using fiber-optic cables leveraging telecommunication infrastructure. Below you can find references to 5 other publications, listed in chronological order that I cut and pasted from the web. These publications report about two other data-collections experiments that use fibre-optic cables placed in telecommunication conduits. Please notice that the first one was actually published a few weeks before the first reference describing the experiment reported in this paper. I think that a subset of these publications should be referenced in the paper.

Thanks to point out those references to un-reviewed papers or conference. As suggested by the reviewer, we include some of them (references 37 and 38). We note that our study was also reported in similar publications that we also report here (references 34 and 35).

We would like to thank again the reviewers and the Editor for their comments and suggestions and do hope the manuscript is now suitable for publication in Nature Communications.

Philippe Jousset
And the other co-authors.

Editorial Note: Author Responses to Reviewer 2 comments on manuscript file

To the comment on line 99: “The cable was trenched with plough and covered with soil and rocks according to information we received from the telecom company. We modified the text accordingly and added a reference where the details of the coupling are described.”

To the comment on line 163: “We agree that we have two possible solutions in case of a straigth 1-D array of receivers. However, the cable we used in Iceland is curved. Therefore, the receivers form a non linear distribution in space leading to a unique solution for the localisation. The text was modified to specify this. A supplementary figure is added.”

To the comment on line 170: “We believe the reviewer refers to Fig 5 iinstead of Fig 4d. We disagree with reviewer 2 here. Although the velocity is low, we observed that the microseism (5-6s period) and above also trapped and possibly bouncing (Supplementary Figure). Therefore it cannot be reflections in air. In addition, the topography is flat without reflectors at the surface. We interpret our observations by the fact that the damage zone in a fault is indeed very loose and velocities can be very low. However, we agree that probably the surface properties of the fault zone can be retrieved only. therefore we added "subsurface" to the text.”

To the comment on line 194: “Figure 2c is not corrected for amplitude and phase response (see text for explanation). Therefore, only relative amplitudes that are not corrected for the instrumental response of the DAS system can be compared. As the phase response of the DAS system is constant in the frequency range of interest, phases can be compared. An additional panel was included.”

To the comment on Figure 3, Page 10: “Acknowledged. The figure was modified.”

To the comment on line 287: “The purpose of this sentence was not clear enough. Therefore we rephrased it.”

To the comment on line 299: “Please see reply to comment on line 163. The cable is curved. Please see suppelmentary material as well. A reference to the supplementary material was included.”

To the comment on line 322: “Acknowledged!”

To the comment on line 341: “Acknowledged. Text was modified accordingly.”

To the comment on line 349: “Acknowledged! We changed the text to "towards imaging"”

To the comment on line 358: “See our reply to previous comment. We added a reference here..”

To the comment on line 365: “You are right that this is close to the velocity of air biut please refer to our previous argument about the microseism as well.”

To the comment on line 406: “Acknowledged. We removed that sentence and added reference in the introduction.”

To the comment on line 414: “See previous replies.”

To the comment on line 415: “See previous replies. We added a sentence to exclude the effect of waves travelling through air, here.”

To the comment on line 430: “We rephrased the text here. Thank you for spotting this.”

To the comment on Figure S1, page 38: “This is for a 10m gauge length, i.e. for the DAS onfiguration used in the field.”

To the comment on line 930: “Acknowledged. We refer to an apparent ground velocity and an apparent wavelength.”

To the comment on line 940: “Acknowledged.”

To the comment on line 986: “You are right, this is misleading. Text was rephrased.”

To the comment on Figure S8a, page 43: “Acknowledged.”

To the comment on line 1002: “Text has been modified.”
